# Germanium in Carbon Fullerenes: Quantum-Chemical Insights into Substitution, Adsorption, and Encapsulation Phenomena

**DOI:** 10.3390/ijms262412067

**Published:** 2025-12-15

**Authors:** Monika Zielińska-Pisklak, Adrianna Jakubiec, Łukasz Szeleszczuk, Marcin Gackowski

**Affiliations:** 1 Department of Pharmaceutical Chemistry and Biomaterials, Faculty of Pharmacy, Medical University of Warsaw, 1 Banacha Str., 02-093 Warsaw, Poland; 2 Department of Organic and Physical Chemistry, Medical University of Warsaw, 1 Banacha Str., 02-097 Warsaw, Poland; s088865@student.wum.edu.pl (A.J.); lukasz.szeleszczuk@wum.edu.pl (Ł.S.); 3 Department of Toxicology and Bromatology, Faculty of Pharmacy, L. Rydygier Collegium Medicum in Bydgoszcz, Nicolaus Copernicus University in Torun, 2 Jurasza Str., 85-089 Bydgoszcz, Poland; marcin.gackowski@cm.umk.pl

**Keywords:** germanium-doped fullerenes, heterofullerenes, substitutional doping, germylation, endohedral metallofullerenes, exohedral complexes, DFT, electronic structure, adsorption, nanomaterials, carbon nanostructures

## Abstract

Germanium (Ge) incorporation profoundly modifies the structural and electronic characteristics of carbon fullerenes, giving rise to a diverse landscape of substitutional, exohedral, and endohedral Ge–fullerene architectures. Although experimental studies demonstrate that Ge can be introduced into fullerene matrices through nuclear recoil implantation and arc-discharge synthesis, only exohedral germylated derivatives have been structurally confirmed to date. Substitutional germanium-doped fullerene (Ge-C_60_) species remain experimentally elusive, with available evidence relying largely on radiochemical signatures and indirect spectroscopic data. In contrast, computational investigations provide a detailed and coherent picture of germanium doping across fullerene sizes, showing that Ge induces significant cage distortion, breaks local symmetry, narrows the highest occupied molecular orbital–lowest unoccupied molecular orbital (HOMO–LUMO) gap, and enhances charge localization at the dopant site. These electronic perturbations strongly increase the affinity of Ge-doped fullerenes for external guest molecules, leading to enhanced adsorption energies and distinct optical and transport responses in exohedral complexes. Theoretical studies of endohedral systems further indicate that Ge atoms or small clusters could form stable encapsulated species with unique electronic properties. Collectively, current evidence positions germanium-doped fullerenes as electronically versatile nanostructures with potential applications in sensing, optoelectronics, catalysis, and nanomedicine, while highlighting the need for definitive experimental synthesis and structural validation of substitutional Ge-fullerene derivatives.

## 1. Introduction

### 1.1. Fullerenes: Structure, Properties and Technological Relevance

Carbon nanomaterials represent one of the most versatile and influential classes of modern functional materials. Their unique combination of electronic conductivity, high surface area, tunable chemistry and nanoscale structural diversity has driven major progress in fields such as electronics, energy storage and conversion, catalysis, drug delivery and molecular medicine [1,2]. Among them, fullerenes occupy a special position as closed-cage carbon allotropes with exceptional stability, rich redox chemistry and a delocalized π-electron network that enables diverse physical and chemical interactions [3]. These properties have led to wide exploration of fullerene-based materials in organic photovoltaics, superconductors, nanocarriers in biomedicine, contrast agents, and quantum electronic devices [4,5]. Building on this relevance, molecular modification of fullerene cages—including heteroatom substitution and endohedral or exohedral functionalization—offers powerful strategies for tailoring electronic structure and reactivity toward targeted applications [6].

Among carbon nanostructures, fullerenes—also known as Buckyballs—stand as the prototypical example of a hollow, closed-cage carbon framework [1]. Their truncated icosahedral architecture, high icosahedral symmetry (I_h_), and remarkable thermodynamic stability make them one of the most iconic nanostructures in carbon science [2]. A distinctive feature of fullerenes is the presence of an internal cavity, which enables them to function as nanoscale containers capable of encapsulating atoms, ions or small molecular clusters [3,4,5,6]. This combination of structural rigidity, electronic delocalization and internal volume underlies their application in molecular storage, endohedral complexes, drug delivery vectors and quantum electronic systems—firmly establishing fullerenes as foundational building blocks for functional nanotechnology [1,2,3,4,5,6].

### 1.2. Structure, Variants and Doping Strategies in Fullerene Chemistry

Fullerenes constitute a unique class of carbon allotropes characterized by closed-cage structures composed of fused pentagonal and hexagonal rings [1]. The archetypal fullerene, C_60_, often referred to as buckminsterfullerene, was discovered in 1985 through laser vaporization of graphite and is renowned for its truncated icosahedral geometry, which closely resembles a soccer ball or a geodesic dome structure [1,2]. C_60_ possesses icosahedral symmetry and displays a range of remarkable physical and chemical features, including high electron affinity, extensive π-electron delocalization, and pronounced thermodynamic stability [3,4]. Each carbon atom in C_60_ is bonded to three neighbors in an sp^2^ hybridization, resulting in a versatile electronic structure that facilitates a variety of chemical transformations, most notably addition reactions rather than reactions characteristic of classical aromatic systems [5,6]. In addition to C_60_ (a fullerene composed of 60 carbon atoms), the fullerene family encompasses smaller and larger carbon cages such as C_20_ (20 C atoms), C_28_, C_70_, and C_84_, each named according to the number of carbon atoms forming the cage, with each comprising even numbers of carbon atoms interconnected to form polyhedral cages [7,8,9,10]. C_20_ is the smallest, but exhibits high internal strain and reactivity due to its all-pentagonal topology [11,12,13,14]. Larger fullerenes like C_70_, C_76_, C_84_, and upwards feature elongation and structural diversification, incorporating more hexagonal rings while keeping the canonical 12 pentagons essential for closure and topology in accordance with Euler’s theorem [15,16]. These size variations lead to a broad range of electronic and structural properties, with potential applications extending from materials science to biomedicine and optoelectronics [17,18,19,20,21].

Beyond pristine carbon cages, further tunability of fullerene properties can be achieved through heteroatom substitution (doping) [22,23,24]. Introducing foreign atoms into the fullerene framework—such as boron, nitrogen, silicon, or germanium—alters the electronic structure, stability, and reactivity of the cages [25,26,27,28,29]. In particular, germanium-doped carbon fullerenes represent a distinct class of heterofullerenes, where substitutional Germanium (Ge) atoms induce characteristic modifications in geometry and electronic behavior, opening new perspectives for functional applications [30,31,32]. Among the possible heteroatom substitutions in carbon fullerenes, germanium represents a particularly intriguing case. Being a group-14 element like silicon, Ge possesses a comparable valence electron configuration but exhibits a larger covalent radius (≈122 pm vs. ≈111 pm for Si) and lower electronegativity (2.01 vs. 2.55 on the Pauling scale) [33,34,35]. These differences imply that Ge substitution within the fullerene cage is likely to induce local geometric distortions, such as elongated C–Ge bond lengths relative to C–C, and may also reduce cage symmetry; analogous geometric perturbations have been observed experimentally for Ge-implanted graphene [36]. Furthermore, the electronic perturbation introduced by Ge atoms alters the π-electron delocalization and opens an energy gap in the electronic structure, as demonstrated for Ge-doped graphene, where the substitution leads to semiconductor behavior and significant modification of optical and electronic properties [37]. This effect is consistent with theoretical studies highlighting the strong impact of group-14 doping (including B, Si, Ge) on the structure, conductivity, and electronic transitions in carbon-based nanostructures [38].

In the context of fullerene modification, germanium can interact with carbon cages through three distinct structural motifs, each associated with different bonding environments and electronic consequences:

(i) Substitutional doping (Ge–C_n_ heterofullerenes):

A Ge atom replaces one of the carbon atoms within the fullerene framework, forming Ge–C bonds and causing local cage distortion. This modification perturbs π-electron delocalization and often narrows the HOMO–LUMO gap [26,27,28,29,30].

(ii) Exohedral adsorption (surface-bound Ge):

Here, Ge resides outside the fullerene cage, interacting with the carbon surface via chemisorption or physisorption. Such Ge-on-cage complexes enhance molecular polarity and improve affinity toward external molecules, enabling guest binding, catalysis, or sensing applications [31,32,33,34].

(iii) Endohedral encapsulation (Ge@C_n_):

In this configuration, Ge is located inside the hollow fullerene cavity, forming endohedral metallofullerenes. Encapsulation can significantly alter electron distribution and magnetic properties, though experimentally these structures are challenging to isolate [23].

Together, these three modes define the structural landscape of germanium-fullerene systems and provide a framework for understanding the computational and experimental results discussed in subsequent sections of this review.

In recent decades, quantum-chemical computations have become essential for elucidating the properties of fullerenes and their heteroatom-doped derivatives. Methods such as DFT and ab initio molecular dynamics allow reliable prediction of cage geometries, bond alternation, electronic delocalization, vibrational spectra, and energetic stability. For heterofullerenes, theory is especially vital: it quantifies how substitution or complexation perturbs local symmetry, modifies HOMO–LUMO gaps, dictates charge redistribution, and screens plausible isomers before synthetic attempts. Indeed, systematic DFT studies—e.g., on C_59_X cages where X = B, N, Si, Ge—have mapped trends in stability, electronic structure and vibrational behavior across dopants [39]. More generally, reviews on quantum-chemical approaches in fullerene chemistry underscore how computational insights now guide experiment in design of new nanostructures [40].

The present review continues our interest in heteroatom-doped fullerene systems, following our earlier survey on silicon-substituted C_60_ derivatives [41], with the current focus directed toward germanium-containing fullerene architectures. The scope of this article is intentionally wide, encompassing not only the classical substitutionally Ge-doped fullerenes, where carbon atoms of the cage are replaced by Ge, but also endohedral systems, in which germanium atoms or clusters are trapped inside fullerene cages, and exohedral derivatives involving germanium or other atomic and molecular guests bound externally to the cage surface. By analyzing these diverse motifs in different cage sizes (C_20_, C_60_, C_70_, C_84_ and larger), this review aims to provide a systematic description of how germanium incorporation modifies the structure, stability and electronic properties of carbon fullerenes. In scientific literature there is an extensive body of work on pure germanium clusters and related cage-like nanostructures, such as Ge94^−^ units, Ge24 clusters, clathrate frameworks, and functionalized nanocages, studied both theoretically and experimentally [42,43]. Numerous studies describe also transition-metal doped or endohedral germanium clusters (e.g., M@Ge_12_, M = Co, Pd, Zr, Nb) with emphasis on stability, electronic structure, and potential applications [44,45,46,47], as well as heteroatomic Ge-based nanoclusters including Ge-B cages, Ge-Si frameworks, and mixed endohedral complexes [48,49]. Although these systems form an active and rapidly developing area of cluster science with implications for catalysis, optoelectronics, and energy storage, they fall outside the scope of this review, which is devoted specifically to carbon fullerenes modified by germanium. Readers seeking detailed information on pure germanium clusters are referred to the references cited above.

In summary, this review provides a broad perspective on Ge-doped carbon fullerene chemistry, including substitutional, endohedral, and exohedral derivatives as well as hybrid forms. By combining computational and experimental insights, it maps the current state of the field, highlights open questions, and discusses potential applications in nanotechnology, optoelectronics, catalysis, and biomedicine.

## 2. Attempts Toward Synthesis of Ge-Doped Fullerenes

Experimental efforts to incorporate germanium into carbon fullerenes have been pursued using diverse approaches, ranging from nuclear recoil implantation and arc-discharge synthesis to photochemical and organometallic functionalization. Although germanium—fullerene systems are far less explored than their silicon counterparts, several landmark studies have provided direct or indirect evidence of Ge substitution, adsorption, and encapsulation in fullerene frameworks.

### 2.1. Traditional Synthesis: High-Energy Methods (Arc Discharge & Laser Vaporization)

The first experimental indication of germanium incorporation into fullerenes was reported by Ohtsuki et al., who investigated the formation of As- and Ge-doped heterofullerenes via nuclear reactions involving C_60_/C_70_ targets mixed with GeO_2_ powders. Following deuteron irradiation, nuclear recoil implantation produced radioactive species identified as GeC_59_ and related polymeric forms. Gamma-ray spectroscopy confirmed the presence of germanium in fullerene-derived species, but could not unambiguously assign whether Ge was incorporated endohedrally, substitutionally, or remained surface-associated. Radiochemical and radiochromatographic analyses further demonstrated that ^69^Ge-labeled fullerene monomers, dimers, trimers, and tetramers are present in the soluble fraction. Ab initio molecular-dynamics simulations further suggested that substitutional incorporation is energetically plausible under recoil conditions [50]. Building on these results, Ohtsuki and Ohno extended their radiochemical approach using p- and d-induced recoil implantation reactions on C_60_/GeO_2_ composites. Gamma-ray spectroscopy and classical molecular-dynamics simulations, indicated the presence of Ge incorporated into fullerene matrices, with signals consistent (but not definitive) with endohedral and/or defect-associated species. However, an unambiguous structural assignment—particularly a definitive substitutional Ge-C_60_ derivative—was not achieved. These pioneering works established the feasibility of germanium incorporation through high-energy nuclear recoil processes, though the yields were extremely low and the separation of products remained challenging [51].

### 2.2. Challenges of Substitution

Further progress in radiochemical encapsulation was made by Saha and co-workers, who employed a recoil-implantation technique using heavy-ion induced nuclear reactions to incorporate various radioactive isotopes into C_60_ films. Among the tested species, ^69^Ge showed particularly high retention (≈73%) in the organic phase, compatible with endohedral encapsulation and enabling a quantitative estimate of Ge uptake. Nonetheless, the differentiation between endohedral, substitutional, and interstitial configurations relied on radiochemical behavior rather than direct structural spectroscopy, and mixed populations of Ge–fullerene species likely coexisted [52].

### 2.3. Spectroscopic Proof of Ge Incorporation

**Figure 1:**
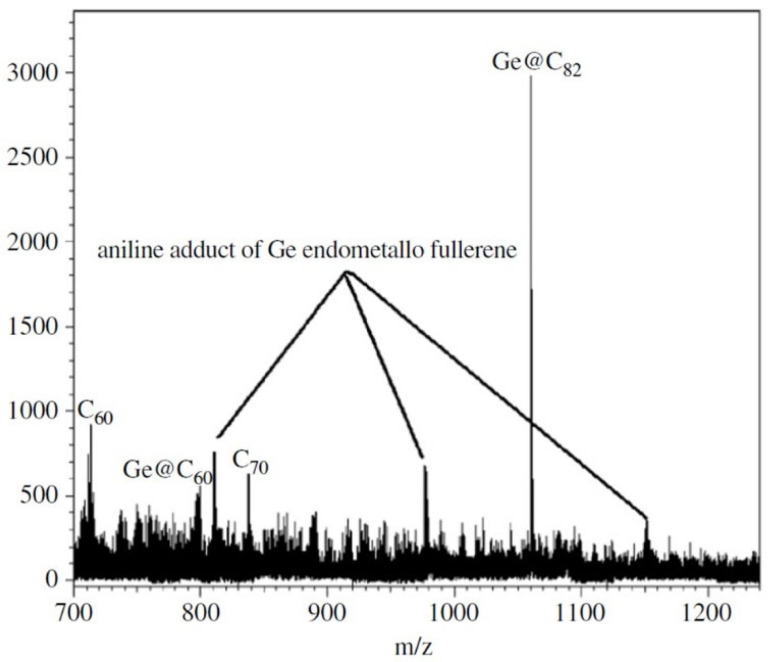
MALDI-TOF spectrum of annealed aniline extract of Ge endometallo fullerene. The intense signal at 1058 *m*/*z* indicates the abundance of Ge@C_82_ in the isolated metallofullerene sample. Reprinted from [32], with permission from Elsevier.

The first successful spectroscopically confirmed arc-discharge synthesis of germanium-encapsulated fullerenes was achieved a decade later by Roy and colleagues. Using composite GeO_2_-graphite electrodes, they generated carbon soot containing Ge@C_60_ and Ge@C_82_ species. Spectroscopic analyses—including UV-Vis, MALDI-TOF, FTIR, XPS, and EDX—supported the endohedral location of Ge rather than external attachment (Figure 1). This study provided the first structural and spectroscopic confirmation of endohedral Ge-fullerenes obtained under arc-discharge conditions, demonstrating that Ge encapsulation can be achieved through conventional fullerene synthesis routes in addition to nuclear recoil implantation methods [32].

### 2.4. Mixed Si–Ge-Doped Analogs and Their Preparation

In addition to monodoped Ge–fullerenes, several studies have explored mixed Si–Ge heterofullerenes, where both group-14 elements are incorporated simultaneously into the carbon cage. These materials are typically formed under high-temperature plasma conditions, where Si and Ge precursors are co-evaporated with graphite, producing C_60−x_(Si,Ge)_x_ clusters detected by mass spectrometry [31]. The presence of both dopants is believed to stabilize the heterofullerene cage through electronic compensation, reducing structural strain compared to pure Ge substitution. Experimental reports describe Si/Ge-codoped species primarily as exohedral or partially substitutional adducts, while isolation of a fully characterized mixed substitutional analog remains challenging. Although structurally confirmed examples are still limited, mixed Si–Ge systems demonstrate a promising route toward multi-heteroatom fullerene engineering [53,54,55,56,57].

Additional arc-discharge experiments conducted by Yılmaz and Uluğ further explored germanium incorporation under helium-atmosphere discharge conditions, using graphite tubes filled with elemental Ge. Their procedure yielded CS_2_-extractable fullerene fractions whose FTIR spectra displayed characteristic C_60_/C_70_ bands, while FAB mass spectrometry revealed a sequence of peaks at *m*/*z* values incompatible with pristine fullerenes, organized in recurring patterns separated by 44 amu increments. The authors interpreted these mass-spectrometric series as evidence for heterofullerene formation, potentially involving Ge substitution and/or exohedral addition of heteroatoms. However, the observed peak multiplicity, the coexistence of C, N and O-containing species, and the lack of elemental analysis or high-resolution structural spectroscopy prevented unambiguous assignment of germanium-containing fullerene derivatives. Thus, while the Yılmaz study supports the possibility of Ge incorporation under arc-discharge conditions, it does not provide definitive structural evidence for specific Ge-fullerene architectures [31].

Complementary mechanistic insight into germanium–carbon interactions was provided by Ohara et al., who investigated the aggregation behavior of Ge atoms and clusters on the C_60_ surface under gas-phase conditions using two-laser vaporization. Their mass spectrometry and photoelectron spectroscopy analyses revealed that small germanium clusters (Ge_1-3_) weakly adsorb onto the fullerene shell, and do not penetrate or substitute the cage under studied conditions. The observed clusters were predominantly stabilized by relatively weak interactions with the surface, as supported by photoelectron spectra and computational results. This work demonstrated that exohedral germylation, rather than endohedral encapsulation, is the preferred mode of germanium incorporation under the studied conditions, thereby clarifying the initial stages of Ge-C_60_ surface chemistry [53].

A more defined class of exohedral Ge-fullerenes emerged from photochemical reactions. Akasaka and co-workers reported the first bis-germylation of C_60_ with a digermirane under photolytic conditions, yielding a well-defined 1,4-cycloadduct featuring two Ge-C bonds on opposite cage sites. The reaction proceeded via a photoactivated exciplex intermediate and produced well-defined adducts characterized by NMR and UV-Vis spectroscopy [54]. Shortly thereafter, Kabe et al. described a related photochemical reaction of 1,2-digermacyclobutane with C_60_, producing an isolable germylated derivative incorporating [6,5]- and [6,6]-bridging units. PM3 semiempirical calculations supported a closed-cage [6,5] structure featuring a Ge-C bridge, representing one of the earliest examples of controlled exohedral Ge-fullerene bonding [55].

Subsequent studies extended germylation to endohedral metallofullerenes (EMFs). Kako et al. successfully prepared and structurally characterized bis-germylated Lu_3_N@Ih-C_80_ derivatives formed photolytically from digermiranes. X-ray diffraction and electrochemical analyses revealed that Ge substitution lowered redox potentials and influenced the localization of the encapsulated metal cluster [56]. This work was later complemented by a comprehensive review by Kako and colleagues, summarizing the functionalization chemistry of fullerenes and EMFs with reactive silicon and germanium compounds. The authors highlighted that germylation not only alters HOMO–LUMO energy levels but also enhances electron-donor capacity, suggesting potential for electronic and catalytic applications [57].

Collectively, these pioneering studies define the current experimental landscape of Ge-fullerene chemistry. A comparative synopsis of their methodologies, analytical tools, main findings, and limitations is presented in Table 1. Taken together, these studies establish the presence of Ge within fullerene matrices (radiochemical/arc-discharge) and on cage surfaces (photochemical germylation), while structurally authenticated substitutional Ge-C_60_ derivatives remain unverified. Numerous DFT and ab initio studies predict their stability, bond metrics, and electronic modifications, but no reproducible experimental synthesis or isolation of substitutional Ge-C_60_ derivatives has been reported [39,58,59,60].

Alongside the germanium-focused studies reviewed above, a substantial body of work on well-characterized endohedral fullerenes—such as i.e., Li@C_60_, N@C_60_, and related fulleride species—provides important methodological benchmarks for assessing the structural validity of proposed Ge-fullerene complexes. Early demonstrations of atom-in-cage architectures, such as the encapsulation of atomic nitrogen (N@C_60_) reported by Lips et al. [61], established foundational criteria for identifying genuine endohedral species using EPR/ESR signatures and stability measurements. Subsequent advances, including precise structural determination by Aoyagi et al. [62], controlled redox-state manipulation and isolation protocols reported by Ueno and colleagues [63,64], and mechanistic insights into switching behavior demonstrated by Chandler et al. [65], illustrate the analytical standards typically required to establish atom-in-cage architectures with confidence. Complementary synthetic achievements demonstrate the necessity of reproducible separation and crystallographically supported structural assignment to validate endohedral doping [66]. Parallel developments by Neyts and co-workers, and mechanistic studies of endohedral metallofullerene formation [67], highlight the necessity of convergent evidence from complementary techniques—such as single-crystal X-ray diffraction, EPR/ESR spectroscopy, systematic electrochemistry, and reproducible spectroscopic characterization—to differentiate endohedral encapsulation from exohedral binding or surface association. Collectively, these studies establish the analytical standards and methodological expectations that inform the interpretation of germanium-fullerene experiments, where structural assignments are inherently more challenging due to low yields and indirect detection pathways. The established structural benchmarks for N@C_60_ and Li@C_60_ have not yet been achieved for any substitutional germanium fullerene; all assignments for Ge rely thus far on indirect, rather than direct structural or spectroscopic, evidence.

Against this established analytical background, the current germanium-fullerene literature remains limited in terms of definitive structural assignments. Radiochemical recoil implantation experiments demonstrate germanium incorporation into fullerene matrices but do not resolve atomic positions within specific molecular species [50,51,52]. Arc-discharge synthesis provides spectroscopic signatures consistent with the presence of Ge inside fullerene cages, yet without direct structural determination [31,32]. Photochemical germylation reactions reliably yield exohedral Ge-C_60_ derivatives, but do not access substitutional or encapsulated configurations [54,57]. As a result, while germanium involvement in fullerene-derived species is supported experimentally, the precise structural roles of Ge-substitutional, exohedral, or endohedral-remain only partially established, and no structurally authenticated substitutional Ge-C_60_ derivative has so far been reported [39,58,59,60].

This situation emphasizes the complementary importance of computational approaches. Quantum-chemical studies offer a consistent and detailed description of the structural, electronic, and energetic consequences of germanium incorporation into carbon fullerenes, including substitutional, exohedral, and endohedral motifs. In many cases, theoretical models provide the only available insight into stability trends, charge redistribution, orbital hybridization, and potential reactivity. Consequently, the next section of this review focuses on the current state of in silico investigations, summarizing the most informative computational methodologies and the principal trends they reveal across Ge-C_n_ systems.

## 3. Germanium Incorporation: The Three Modes

Germanium can interact with and integrate into fullerene cages through three fundamentally distinct structural pathways, each associated with a unique bonding environment, stability profile and electronic response. These modes are summarized below.

### 3.1. Substitution (Germanium as a Cage Member)

In the substitutional configuration, a carbon atom in the fullerene cage is replaced by a germanium atom, forming a heterofullerene of the type C_60−1_Ge [39,68,69,70]. This process introduces a heteroatom directly into the π-conjugated network, modifying local curvature and rehybridization. Substitutional incorporation significantly perturbs delocalized electrons and typically narrows the HOMO–LUMO gap [39,69,71]. However, the synthetic realization of substitutional Ge–fullerenes remains highly challenging, with no crystallographically authenticated C_59_Ge derivative reported to date [39,70,71,72,73,74,75,76,77,78,79].

### 3.2. Adsorption (Exohedral Interaction)

In the adsorption (exohedral) mode, Ge binds to the external surface of the fullerene cage, forming a Ge–C interaction without breaking the cage framework. These complexes are more synthetically accessible and can be produced via photochemical germylation or surface chemisorption routes [54,55,56,57]. Exohedral attachment modifies electronic distribution and often increases dipole moment, enhancing reactivity toward donor/acceptor molecules and catalytic interfaces [80,81,82,83,84,85,86,87,88,89,90,91,92,93,94].

### 3.3. Encapsulation (Endohedral Containment)

In the encapsulation mode, Ge is located inside the internal cavity of the fullerene cage, forming Ge@C_n_ endohedral metallofullerenes [31,32,50,51,52]. Encapsulation may occur under arc-discharge or recoil implantation conditions where high energy promotes atom insertion through cage opening. These structures show remarkable electronic rearrangement, but direct structural proof remains sparse, with only spectroscopic evidence currently supporting Ge@C_60_/Ge@C_82_ species [31,32].

## 4. Quantum-Chemical Characterization of Ge-Fullerenes

Following the limited but informative experimental attempts to generate Ge-containing fullerene derivatives, quantum-chemical investigations have emerged as the principal route for understanding their structural, electronic, and bonding characteristics [27,39]. Because experimentally accessible Ge-fullerenes are produced in extremely low yields, often as mixtures of short-lived species detectable only by indirect radiochemical or spectroscopic methods, ab initio and density functional theory (DFT) calculations provide the resolution required to examine germanium incorporation with atomic-level precision. Consequently, computational approaches have become indispensable for exploring the substitutional, exohedral, and endohedral motifs proposed in experimental studies [40].

Theoretical investigations have addressed a series of core questions, including the energetic feasibility and relative stability of different germanium-doping patterns, the extent to which Ge atoms induce symmetry breaking and structural distortions in the fullerene cage, and the consequent modifications in aromaticity, curvature, and overall bonding topology. Particular attention has also been devoted to quantifying charge transfer between germanium and the carbon framework, as well as assessing how such electronic redistribution influences the HOMO–LUMO separation, frontier-orbital localization, and magnetic response of the resulting heterofullerenes [69].

The following sections synthesize the major computational findings for substitutionally germanium-doped, exohedral, and endohedral germanium-fullerene systems. Emphasis is placed on methodological aspects—such as functional and basis-set choices, geometry optimization protocols, ESPA analyses, and dynamical simulations—and on the insights these approaches provide into the stability, electronic structure, and reactivity of Ge-C_n_ heterofullerenes.

### 4.1. Substitutionally Ge-Doped Fullerenes

Substitutional incorporation of germanium atoms into carbon fullerenes has been extensively investigated through ab initio and density functional theory calculations as a means of tuning the intrinsic electronic and structural characteristics of carbon cages. In these heterofullerenes, one or more carbon atoms are replaced by germanium, leading to local expansion of the cage (C-Ge ≈ 1.82–1.90 Å), symmetry reduction, and polarization of the π-electron density. These effects collectively modify the stability, aromaticity, charge distribution, and electronic transitions of the system [70,71]. Computational studies have examined a wide variety of substitutional Ge-fullerenes, encompassing C_20-n_Ge_n_ systems with varying degrees of substitution (n = 1–10) [60,72,73,74,75,76], as well as larger cages such as C_58_, C_59_, C_69_, and C_70_ [39,50,69,70,71,77,78,79].

Mixed Si–Ge-doped analogs of C_20-n_(Si,Ge)_n_ have also been explored to assess cooperative effects of co-doping on electronic polarization and cage distortion [80,81]. Theoretical modeling consistently showed that Ge substitution narrows the HOMO–LUMO gap, increases the dipole moment, and induces charge localization near the dopant site [60,70,73,74,77]. For low-substituted C_20-n_Ge_n_ systems, incorporation of one or a few Ge atoms leads to localized cage distortion and polarization, whereas in higher substitution regimes (e.g., n ≥ 6) or in larger cages such as C_69_ and C_70_, the deformation becomes more delocalized across the fullerene surface, with smoother redistribution of π-electron density [60,77].

A wide spectrum of computational protocols has been used to characterize these systems. The B3LYP functional combined with split-valence polarized basis sets such as 6-31G(d), 6-311+G(d), or 6-311++G(d,p) remains the most widely adopted choice [39,60,71,72,73,74,76,79,80,81]. Other functionals, including B3PW91, M06-2X, and dispersion-corrected PBE-D3, have been applied to benchmark electronic energies and bond-length deviations [60,77,81]. Most calculations were performed using Gaussian 98/09 [39,71,72,73,74,75,79,81], DMol^3^ [77], GAMESS [60,76,80], HyperChem [78], or MOPAC (CAChe) [69], while Atomistix ToolKit (ATK) and Virtual NanoLab were employed for transport and device-level simulations [82].

The majority of computational studies involved geometry optimization (GO) and electronic structure and property analysis (ESPA), often complemented by vibrational frequency analysis (VFA), and NICS aromaticity evaluation [39,60,69,72,73,74,76,79,80,81]. The results consistently indicated significant charge transfer from Ge to adjacent carbon atoms, accompanied by partial loss of aromatic character and a reduced HOMO–LUMO separation [39,69,72]. For structures such as C_59_Ge, and C_69_Ge, DFT analyses revealed that substitution sites with higher local curvature are thermodynamically preferred and that Ge substitution notably enhances molecular polarity and dipole moment [70,71]. Smaller germanium-doped cages (e.g., C_12_Ge_8_, C_14_Ge_6_, C_19_Ge) exhibit stronger cage strain but retain substantial cohesive energy, attributed to σ-rehybridization between Ge and neighboring carbons [60,73,74]. In contrast, larger derivatives such as C_70-n_Ge_n_ or C_20_-C_16_Ge_4_ display lower deformation energy and enhanced delocalization, consistent with increased structural flexibility of extended carbon frameworks [77,82].The most interesting applications of calculations in the study of these structures are presented below.

**Figure 2:**
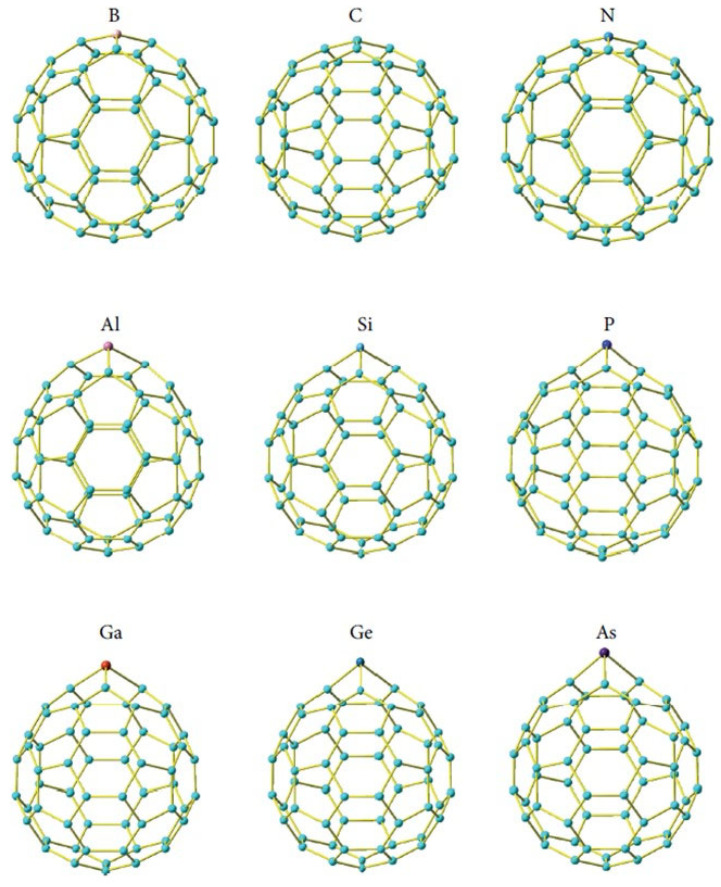
Structures of C_59_X (X = B, N, Al, Si, P, Ga, Ge, and As) and C_60_ cage. Adapted from [39], licensed under CC BY 3.0.

Bai et al. [39,72] provided one of the earliest systematic DFT analyses of substitutional Ge-doping in C_60_, focusing specifically on the C_59_Ge derivative as part of a broader comparative study of C_59_X (X = B, N, Al, Si, P, Ga, Ge, As). Their results showed that replacing a single carbon atom with germanium leads to a pronounced increase in local bond lengths and measurable cage distortion, consistent with the comparatively large covalent radius of Ge. Importantly, within the full series of dopants investigated, Ge belonged to the subgroup (Ga, Ge, As) that produced the largest sphericity-parameter (SP) values and the largest C-X bond elongations, indicating that Ge induces substantially stronger geometric deformation than lighter group-14 dopants such as Si or than second-row elements such as B or N (Figure 2). In terms of electronic structure, Bai et al. [39,72] demonstrated that C_59_Ge exhibits a significantly reduced HOMO–LUMO gap relative to pristine C_60_, and that this gap narrowing is comparable to that observed for other heavy dopants (Ga, As) but more pronounced than in cages doped with lighter elements.

Their frontier-orbital analysis further revealed that Ge contributes markedly to both HOMO and LUMO densities (≈35.3% and ≈22.4%, respectively), making it one of the most strongly orbital-localizing dopants in the entire C_59_X series. Taken together, these findings position Ge as one of the dopants that most strongly perturbs both the geometry and the π-electron distribution of C_60_, offering a clear internal comparison—based on a single methodology—of how group-III, -IV and -V elements differ in their influence on fullerene properties [39]. Building on these conclusions for the C_60_ framework, Bai et al. [39,72] extended their analysis to the smallest classical fullerene, C_20_, by examining the full C_19_X series, including the C_19_Ge derivative. In contrast to the moderately strained C_59_X cages, the C_20_ scaffold—composed exclusively of adjacent pentagons—exhibits intrinsically high curvature and low symmetry, making it far more sensitive to dopant-induced perturbation. Within this highly constrained environment, Ge ranks among the dopants that produce the largest geometric distortions, with SP and ASP values comparable to those of Ga, As, and Se, and far exceeding those generated by lighter elements such as B, N, or O (Table 2). The C-Ge bond in C_19_Ge belongs to the longest in the entire C_19_X series, and the associated deformation propagates more strongly across the cage than in substitutionally doped C_60_. Electronic reorganization follows the same periodic trend: Ge lowers the HOMO–LUMO gap more substantially than lighter group-14 or group-15 dopants, placing it among the dopants that most effectively destabilize the small fullerene’s π-system [72].

**Figure 3:**
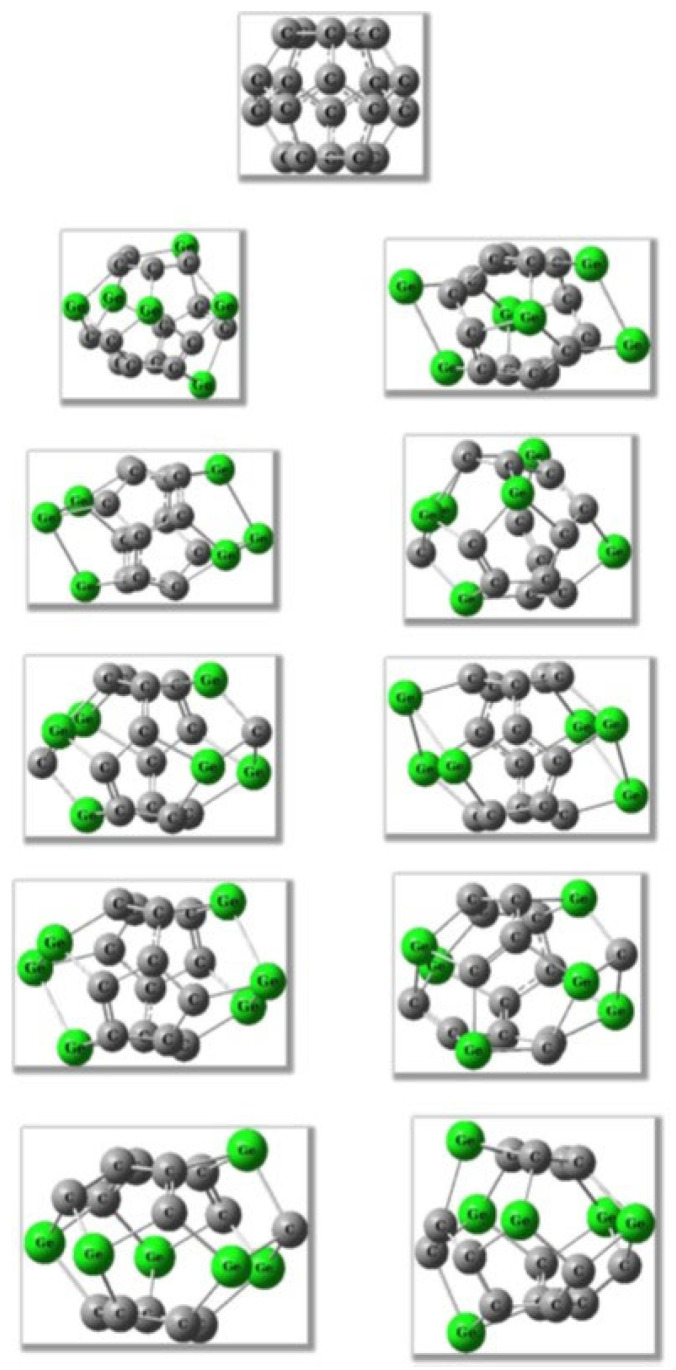
The optimized C_20_ fullerene and its 10 C_14_Ge_6_ analogous. Used with permission from JOHN/WILEY & SONS LTD. [76], permission conveyed through Copyright Clearance Center, Inc.

**Figure 4:**
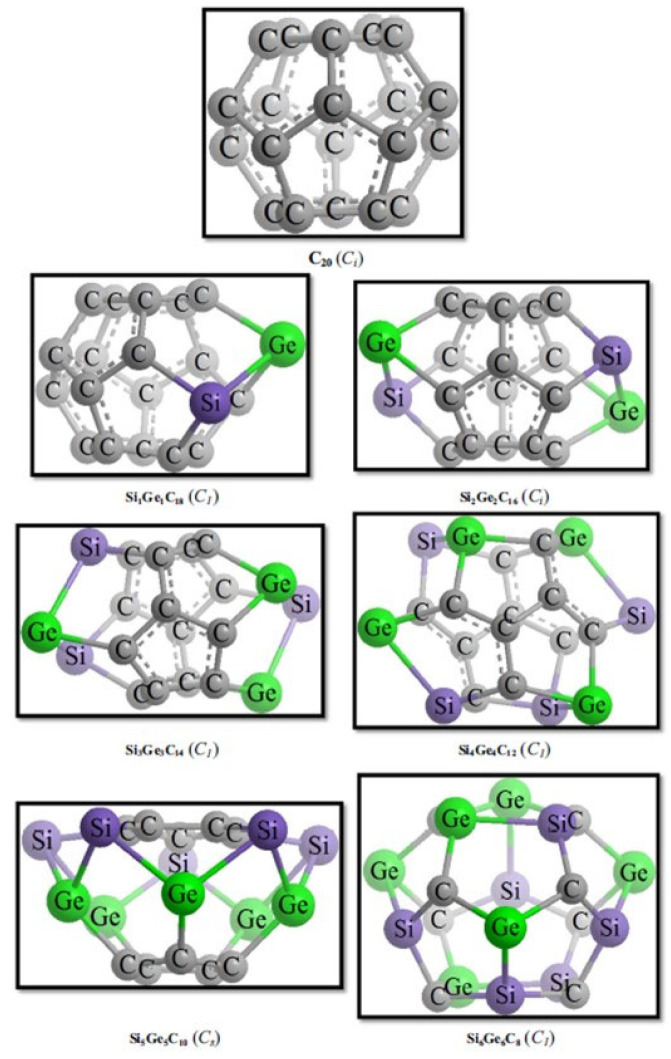
Optimized C_20_ and its C_20-2n_S_n_Ge_n_ derivatives as well as their point groups, at B3LYP/AUG-cc-pVTZ. Used with permission from SPRINGER-VERLAG WIEN [81], permission conveyed through Copyright Clearance Center, Inc.

Extending this comparative perspective, a broader picture of substitutional germanium behavior in small, highly curved cages arises from the systematic studies by Koohi and co-workers [74,76,80,81]. Their work examined extensive families of C_20-n_Ge_n_ heterofullerenes across a wide substitution range (n = 1–10), providing some of the most detailed stability and distortion maps available for Ge-doped carbon frameworks (Figure 3). At low substitution levels (n = 1–4), the C_20_ cage retains its overall integrity, with distortion remaining localized around the Ge atoms—consistent with the trends observed in C_59_Ge and C_19_Ge by Bai et al. [39,72]. However, as germanium content increases (particularly n ≥ 6), the cage experiences rapidly rising skeletal strain, significant polarization, and a systematic reduction in thermodynamic stability, eventually approaching the threshold beyond which the fullerene framework can no longer support extensive heteroatom incorporation. The comparative analyses involving Si-Ge co-doped systems (C_20-2n_Si_n_Ge_n_) reveal that silicon behaves as a less perturbing dopant that induces milder geometric distortions and smaller HOMO–LUMO gap reductions than germanium at matching substitution levels (Figure 4). These results reinforce the broader periodic trend already apparent from Bai’s C_59_X and C_19_X series: silicon preserves cage symmetry more effectively and perturbs the π-framework less strongly, whereas germanium introduces larger bond-length variations, deeper symmetry breaking, and more pronounced electronic localization [80,81].

Complementary insights into high-substitution regimes were provided by Naderi et al., who examined systems such as C_12_Ge_8_ specifically to assess the structural integrity of cages containing large numbers of germanium atoms. Their results demonstrated that, although extensive geometric distortion and symmetry loss occur, the fullerene framework can remain a bound minimum on the potential-energy surface. At the same time, the electronic character of these species undergoes profound reorganization, reflecting a complex interplay between σ-rehybridization around dopant centers and depletion of the π-electron network. In comparison with other heteroatoms examined in the same study (boron (B), aluminum (Al), gallium (Ga), silicon (Si), nitrogen (N), phosphorus (P), and arsenic (As)), germanium consistently occupies an intermediate position: it produces substantially larger geometric perturbations and stronger charge redistribution than lighter group-14 dopants such as silicon, yet does not destabilize the cage as severely as some heavier dopants. Taken together with studies conducted by Koohi and collaborators, these findings delineate how increasing germanium content systematically reshapes stability, electronic structure, and geometric resilience in small, highly curved fullerene cages [73].

Electronic structure modifications induced by germanium substitution have been systematically explored across multiple computational studies. Simeon et al. analyzed C_59_Ge with particular emphasis on frontier-orbital energetics, ionization potential, electron affinity, and local charge redistribution. Their investigation showed that insertion of a Ge atom disrupts the delocalized π-electron network of pristine C_60_, producing pronounced localization of electron density around the dopant site, enhancing molecular polarity, and modifying redox behavior. These findings established clear correlations between dopant-induced charge polarization and changes in electronic response functions [70].

Complementary semiempirical work by Ibrahim et al. on C_60_ doped with Si, Ge, and Al using the PM3 method further demonstrated that Ge substitution leads to systematic enlargement of selected C-C bond lengths, substantial reorganization of the net atomic charge distribution, an increased total dipole moment, and characteristic shifts in the IR-active vibrational modes, with an additional low-frequency band appearing as a direct consequence of the doping process [69].

**Figure 5:**
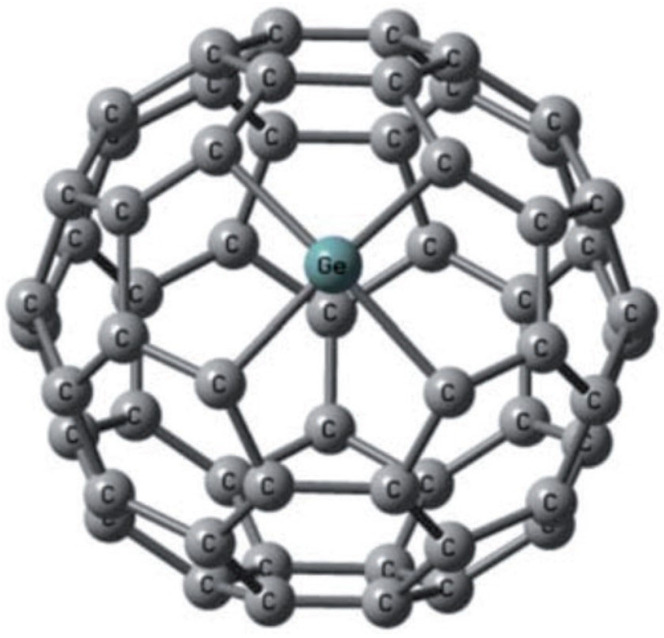
Structure of [5,6]-heterofullerene-like C_58_Ge. Used with permission from ROYAL SOCIETY OF CHEMISTRY [79], permission conveyed through Copyright Clearance Center, Inc.

Ostrowski and co-workers focused on larger heterofullerenes such as C_69_Ge, combining energetic analysis with detailed spectroscopic simulations, including vibrational circular dichroism (VCD), IR, and Raman spectra. Their objective was to delineate how a group-14 heteroatom affects the population of symmetry-distinct isomers and to determine computationally accessible diagnostic criteria for experimental detection. They demonstrated that Ge stabilizes specific low-symmetry isomers by redistributing curvature-induced strain, and they showed that the presence of Ge leads to substantial changes in vibrational signatures. These spectroscopic fingerprints provide potential pathways for distinguishing Ge-substituted cages from pristine fullerenes in complex mixtures [71]. A complementary perspective on substitutional Ge in larger cages was provided by Liu et al., who examined the [5,6]-heterofullerene-like C_58_Ge obtained by substituting a 6-6 C-C bond with a germanium atom. Their DFT (B3LYP/6-31G) and vibrational analyses confirmed that C_58_Ge is a true minimum with C_2_ symmetry, characterized by four Ge-C bonds (≈1.96–2.04 Å), localized distortions in the immediate Ge environment, and an otherwise C_60_-like C-C bond framework; natural bond orbital and frontier-orbital isodensity maps revealed pronounced charge accumulation and orbital participation at the Ge center [79]. A suitable depiction of the optimized [5,6]-heterofullerene-like C_58_Ge cage with emphasis on the local Ge-C bonding environment (Figure 5) illustrates the characteristic distortion pattern around the Ge site in a representative larger heterofullerene.

Beyond ground-state electronic structure, several studies have investigated the impact of germanium substitution on optoelectronic and nonlinear optical (NLO) behavior. Kityk et al. combined theoretical modeling with experimental evaluation of nonlinear optical responses of C_59_Ge and C_58_Ge_2_, correlating specific structural modifications with second-harmonic generation (PISHG) and two-photon absorption (TPA) properties. Their results demonstrated that increased Ge content significantly enhances hyperpolarizability and modulates spectral envelopes, indicating that Ge-induced symmetry breaking directly influences NLO activity [78].

Complementary trends were described by Baei et al., who analyzed C_20-n_Ge_n_ derivatives using frontier-orbital characteristics, aromaticity indices (NICS), and TD-DFT optical simulations. Their goal was to characterize systematic trends in aromaticity loss and band-gap reduction as a function of substitution degree. They found that higher Ge substitution produces consistent red-shifts in optical transitions, increased polarization, and a marked decrease in HOMO–LUMO gaps. These effects underscore the sensitivity of highly curved carbon frameworks to element-specific heteroatom perturbations [60].

The influence of Ge substitution on charge transport has been evaluated in device-relevant computational scenarios. Kaur et al. modeled electron transport in Ge-doped double-cage junctions based on C_20_/Ge-containing C_20_ analogs and demonstrated that Ge incorporation enhances conductance by modifying coherent electron transmission pathways and reshaping the transmission spectrum. These results point toward potential applications of Ge-doped fullerenes in nanoelectronic components, where controlled substitution offers a route to tuning transport characteristics [82].

Alkhafaji with co-workers extended these insights by combining DFT with molecular dynamics simulations to probe finite-temperature behavior, charge distribution, and optoelectronic responses in Ge-doped fullerene systems. Their study revealed that, despite dopant-induced polarization and local symmetry breaking, the fullerene cage retains structural integrity under thermal perturbation and exhibits notable shifts in conductivity profiles. The results illustrate how Ge alters thermal stability and dynamical electronic behavior in ways that may be relevant for functional nanomaterials [77].

Finally, substitutional Ge-doping has been examined in the context of functional interactions. Ajeel et al. investigated C_20_-based Ge-doped structures, focusing on how Ge substitution alters frontier-orbital distribution, electron-donating capability, and redox signatures. In their case, the Ge atoms are introduced into C_20_-derived bowl-like clusters rather than perfectly closed cages, but the qualitative electronic effects mirror those observed for cage-type heterofullerenes: Ge enhances dipole moment, increases electron-donor strength, and intensifies localization of electronic states, suggesting that Ge-doped small C_20_-derived units may exhibit improved sensitivity to external analytes or perturbations. These electronic reorganization effects are consistent with observations in larger cages and provide a unified picture of how germanium modulates reactivity and interaction profiles across different fullerene-sized systems [75]. Selected articles on application of theoretical calculations on substitutional Ge-doped carbon fullerenes are presented in Table 3.

Overall, quantum-mechanical modeling of substitutional Ge-doped fullerenes, encompassing a wide range of cage sizes and substitution degrees, demonstrates that germanium incorporation leads to distinct electronic polarization, symmetry breaking, and localized reactivity patterns. These computational findings provide theoretical benchmarks for understanding the structural behavior, electronic diversity, and potential functional applications of Ge-modified carbon fullerenes in nanotechnology and advanced materials design.

### 4.2. Exohedral Complexes of Substitutional Ge-Doped Fullerenes

Exohedral complexes of substitutionally Ge-doped carbon fullerenes represent an emerging class of heteronanostructures in which external atoms, ions, or molecules bind to the surface of fullerene cages containing one or more substitutional Ge atoms. The incorporation of germanium perturbs the π-electron distribution, enhances local charge polarization, and modifies frontier molecular orbitals, thereby increasing the affinity of the cage toward external guests. These effects manifest as stronger adsorption energies, enhanced charge transfer, and more pronounced modulation of electronic properties relative to pristine fullerenes, positioning Ge-doped fullerenes as promising candidates for sensing, pollutant capture, catalysis, and molecular delivery. Optical simulations reported in recent studies further indicate that Ge substitution can enhance spectral responsiveness upon guest binding [83,84].

Computational investigations reported to date focus predominantly on monosubstituted C_60_-based Ge-doped fullerenes [83,84,85,86,87,88,89,90,91,92,93,94], with only isolated studies extending to higher substitution levels such as C_55_Ge_5_ [95] or mixed heterofullerenes such as Si_11_GeC_12_ [96]. Across these Ge-doped hosts, a wide spectrum of exohedral guests has been investigated. Small organic molecules and environmental pollutants—including acrolein [89] and hexachlorobenzene [88]—were used to evaluate adsorption strength, selectivity, and potential for pollutant scavenging. Biologically relevant small molecules such as serine and cysteine [86,87] revealed substantial charge redistribution and chemisorption behavior, suggesting applications in biosensing. Pharmacologically active compounds—including amphetamine [85], amantadine [83,84], methadone [94], carbamazepine [90], letrozole [91], and natural products such as ferulic acid and EGCG [92,93]—were examined to assess guest–host stabilization, drug–nanocarrier interactions, and solvent-mediated effects. Inorganic ions such as Li [95] were studied to investigate charge transfer, spin-density localization, and metal coordination. Larger heteroatom-containing guests, including caffeine bound to Si_11_GeC_12_ [96] illustrate the versatility of Ge-doped cages for multifunctional adsorption platforms.

The computational protocols across these studies follow a consistent pattern. Nearly all works used geometry optimization (GO) followed by electronic structure and property analysis (ESPA), typically involving HOMO–LUMO analysis, DOS characteristics, NBO-based charge transfer, molecular electrostatic potential mapping, and global reactivity descriptors such as hardness, softness, electrophilicity, and ΔN_max_. Adsorption energies (E_ads_), often with BSSE correction, were widely reported [83,85,86,87,88,89,90,91,92,93,94,96]. Solvation models (PCM/SMD) were also applied to capture environmental effects [83,90,91,96]. Several studies incorporated TD-DFT calculations to analyze optical absorption and sensing-relevant spectral shifts [94,96]. Aromaticity changes upon adsorption were evaluated in selected works using NICS [85]

DFT served as the principal methodological framework. Common functionals included B3LYP [83,94,96], B3LYP-D3 [84,85,86,87,88,89,92,93], PBE0 [86,87,88,89], ωB97X-D [84,86,87,88,89,90], and M06-2X [84,86,87,88,89,90,94], typically combined with split-valence polarized basis sets such as 6-31G(d) [83,90,92,93,94], 6-311G(d) [84,86,87,88,89], 6-311G9(d,p) [91,96] and diffuse-augmented variants like 6-311++G(d,p) [85]. Semiempirical methods such as PM6 or MNDO were used for initial structure screening [86,87,88,89,95]. Gaussian (03/09/16) was the most widely used computational package [83,84,86,87,88,89,90,91,92,93,96], while GAMESS appeared in studies involving hybrid DFT/semiempirical workflows [85,94,95].

The scientific aims of these studies converge on several recurring themes. Many works evaluated adsorption strength, interaction mechanisms, and charge redistribution relevant to sensing or pollutant capture [85,88,89,94]. Others focused on drug-delivery and nanocarrier behavior, assessing solvent effects, stability, and reactivity in Ge-doped drug-fullerene complexes [83,90,91,92]. Studies incorporating TD-DFT and DOS analyses explored optoelectronic signatures relevant to molecular electronics [94,96]. Investigations of metal adsorption addressed charge transport and spin-density localization in substituted cages [95]. Together, these computational contributions delineate the structure-reactivity landscape of Ge-doped exohedral fullerenes and illustrate how substitutional germanium acts as a preferential adsorption center across diverse classes of guest molecules.

The most interesting applications of calculations in the study of these structures are presented below in more detail and Table 4. provides a summary of the computational protocols applied, specifying the semi-empirical methods, DFT functionals, and basis sets used in the respective studies on substitutional Ge-doped carbon fullerenes.

A coherent body of work has explored how substitutional Ge sites modulate the surface reactivity of fullerene cages toward external guests. One of the earliest examples is the study by Bashiri et al. [85], who investigated C_59_Ge as a host for amphetamine adsorption. Their DFT analysis showed that introducing a single Ge atom substantially enhances the interaction strength compared with pristine C_60_, primarily due to increased cage polarization, stronger donor-acceptor coupling, and more pronounced charge transfer. Relative to other doped or pristine fullerenes evaluated in their screening, Ge emerged as one of the strongest adsorption centers, providing the first mechanistic indication that substitutional germanium fundamentally alters surface electronic structure and boosts guest-binding affinity. These findings established C_59_Ge as a sensitive adsorption platform for small, electronically active organic molecules and provided the first mechanistic evidence that substitutional Ge can act as a preferential binding center on the fullerene surface.

Subsequent studies expanded the scope of investigated guests, particularly in the context of antiviral and other pharmacologically active compounds. Parlak and co-workers and Mohammadi et al., working independently on amantadine, demonstrated that adsorption at the Ge site of C_59_Ge produces larger interaction energies, lower HOMO–LUMO gaps, and higher dipole moments than adsorption on pristine C_60_ or on cages doped with other heteroatoms. In these analyses, the Ge atom consistently acted as the electronically dominant interaction site, capable of concentrating frontier-orbital density and mediating stronger donor–acceptor interactions. Taken together, these studies clearly show that substitutional Ge produces stronger adsorption, deeper electronic reorganization, and more pronounced modulation of optoelectronic properties than pristine fullerenes or fullerene cages doped with lighter group-14/15 elements, underscoring the potential of Ge-doped fullerenes as nanocarriers and sensing platforms for small bioactive molecules [83,84].

A complementary perspective emerged from the adsorption studies of Mohammadi et al., who examined biologically relevant amino acids such as serine and cysteine [86,87]. Their results showed that C_59_Ge consistently exhibits stronger adsorption energies than both pristine C_60_ and the Si-doped analog C_59_Si, indicating that the Ge site provides a more polarizable and electronically more responsive adsorption center. In the serine study, the HOMO–LUMO gap followed the order C_60_ > C_59_Si > C_59_Ge, and the adsorption energies became increasingly negative in the same sequence, demonstrating that germanium introduces the strongest donor–acceptor interactions among the tested dopants. QTAIM and NCI analyses further confirmed that noncovalent interactions are more pronounced and spatially more localized around the Ge atom, showing increased electron-density accumulation at the bond-critical points relative to Si-doped and pristine cages. This pattern was reproduced for cysteine, where Ge again produced the most significant charge redistribution and molecular polarization, highlighting the heightened sensitivity of the C_59_Ge surface to polar, heteroatom-rich biomolecules.

Environmental and pollutant-related applications were explored in later studies, which focused on reactive or toxic small molecules. Mohammadi’s et al. DFT analyses of acrolein demonstrated that substitutional Ge enhances electrophilicity changes, DOS reorganization, and adsorption energy relative to C_60_, leading to a more substantial modulation of the electronic structure upon binding [89]. These trends became even more pronounced in the adsorption of hexachlorobenzene, where C_59_Ge exhibited the strongest adsorption of all tested surfaces, with an adsorption energy of −1.010 eV at the B3LYP-D3/6-311G(d) level—significantly stronger than both pristine C_60_ and C_59_Si. QTAIM and NCI descriptors confirmed that these interactions remained noncovalent but were electronically more intense and more spatially extended around the Ge site, further establishing substitutional Ge as a highly effective adsorption center [88]. Collectively, these studies position Ge-doped fullerenes as superior adsorption platforms compared with both undoped and Si-doped cages, particularly for hazardous organics and biologically relevant molecules rich in functional groups. The interaction landscape widens considerably in studies involving structurally complex therapeutic molecules.

Silva et al., Behmanesh, and Singh with collaborators investigated carbamazepine, letrozole, and natural bioactive compounds including ferulic acid and EGCG [90,91,92,93]. Across these drug-fullerene systems, all cited studies consistently showed that introducing a substitutional Ge atom into the C_60_ cage strengthens adsorption relative to pristine C_60_ and noticeably alters global and local reactivity descriptors (electrophilicity, chemical hardness, charge transfer), in a manner compatible with more stable guest–host complexes. At the same time, Ge typically occupies an intermediate position within the full dopant series: adsorption on C_59_Ge is systematically stronger than on undoped C_60_, but in most comparative sets Si- and/or Al-doped cages provide the most energetically favorable binding. Solvation effects, explored through PCM/SMD frameworks, further confirmed that Ge-containing complexes remain stable in aqueous environments, demonstrating that even when Ge is not the strongest dopant in absolute energetic terms, it nonetheless yields pharmaceutically relevant chemisorption and preserves the integrity of the complex in polar media; together, these observations support the potential of Ge-doped fullerenes as nanocarriers for pharmaceutical delivery.

Another dimension of functionality is highlighted in the study by Hoseininezhad-Namin et al., who investigated methadone adsorption on C_59_X cages (X = B, Si, Ge) in a dopant-resolved comparison designed for sensor applications. Within this triad of dopants, Ge emerged as the strongest adsorption center, yielding the highest adsorption energies and the largest charge transfer to the fullerene surface, whereas Si and B exhibited progressively weaker binding (with B doping providing advantages in recovery time and sensitivity rather than binding strength) (Table 5). Their TD-DFT calculations further demonstrated that the optical response depends strongly on the dopant identity: Ge substitution generated the most pronounced dopant-induced spectral shifts upon methadone binding, reflecting substantial polarization of the cage. Collectively, these results show that among the tested dopants Ge most effectively enhances both thermodynamic binding and the optical detectability of the adsorbed molecule—properties directly relevant to fullerene-based sensing platforms [94].

Beyond classical C_60_-based cages, more structurally diverse systems have also been considered. Mahdi et al. investigated caffeine adsorption on the mixed Si_11_GeC_12_ heterofullerene, in which a single Ge atom is incorporated into an Si-rich C20 cage. Within the comparative set of Si_12_C_12_, Si_11_BC_12_ and Si_11_GeC_12_, their results showed that all three hosts support chemisorption of caffeine, but the strongest binding is obtained for the purely Si-doped Si_12_C_12_ and the B-doped analog, whereas Si_11_GeC_12_ exhibits slightly weaker—yet still clearly chemisorptive—adsorption energies. At the same time, Ge substitution noticeably modifies the electronic structure of the Si-rich cage, enhancing charge redistribution and altering frontier-orbital profiles compared with undoped Si_12_C_12_. The presence of Ge produced distinct dopant-dependent optical responses in TD-DFT spectra, with shifts and intensity changes that differ from those induced by B-doping, indicating that Ge modulates the electronic environment in ways not accessible to Si alone. These findings suggest that mixed Si/Ge heterofullerenes may offer tunable adsorption and optical-sensing characteristics, with germanium acting as an intermediate perturbation center—stronger than a purely Si cage in terms of polarization patterns, but not providing the maximal adsorption energy within the Si/B/Ge series [96].

Early insights into inorganic adsorption were provided by Chistyakov, who modeled Li binding to C55Y5 clusters (Y = Si, Ge, Sn, B, Al, N, P, and hydride analogs) using semiempirical MNDO calculations. These studies revealed clear periodic trends: for bare C55Y5 clusters, Ge substitution increases electron density on the substituted pentagonal face and shortens selected C-C bonds relative to unsubstituted C_60_, while for LiC55Y5 η^5^–η^6^ complexes the Li-pentagon bond energy grows in the sequence X = C < Ge < Sn < Si. In other words, Ge significantly enhances Li-pentagon binding compared with pristine C_60_, but remains less stabilizing than Si in this group-14 series. For group-13 dopants (B, Al), Li-pentagon stabilization is even larger, whereas N- and P-doped systems display Li-pentagon bond energies comparable to the carbon reference. These results show that substitutional Ge meaningfully alters the electronic landscape of carbon cages and facilitates metal coordination motifs not accessible in undoped fullerenes, placing Ge as an intermediate-strength stabilizer—stronger than the carbon and several group-15/14 analogs, but weaker than the most strongly binding Si- and Al-doped cages [95].

Taken together, these studies reveal a consistent mechanistic picture in which substitutional Ge acts as a reactive, electronically dominant surface site capable of stabilizing a wide range of external guests. Whether interacting with pharmaceutical molecules, amino acids, pollutants, or inorganic ions, Ge-doped fullerenes exhibit enhanced polarization, stronger charge transfer, and distinctive electronic and optical signatures that differentiate them from both pristine and alternative heterodoped cages. This growing body of work establishes substitutional germanium as a versatile modulator of exohedral fullerene chemistry, expanding the functional landscape of carbon nanostructures across sensing, catalysis, environmental remediation, and drug-delivery contexts.

### 4.3. Endohedral Complexes of Substitutionally Ge-Doped Fullerenes

Endohedral complexes formed within substitutionally doped fullerenes constitute a specialized class of host-guest systems in which a heteroatom embedded in the carbon framework modifies the curvature, symmetry, and electronic landscape of the cage, while an independent atomic or molecular species occupies the interior cavity. This dopant-induced perturbation shapes the internal electrostatic potential and orbital topology, creating an asymmetric confinement environment fundamentally distinct from that of pristine endohedral fullerenes. Such architectures offer a platform for probing how heteroatom substitution influences encapsulation energetics, charge redistribution, and host–guest coupling, with implications for hydrogen storage, molecular electronics, spin-active materials, and quantum nanodevices.

**Figure 6:**
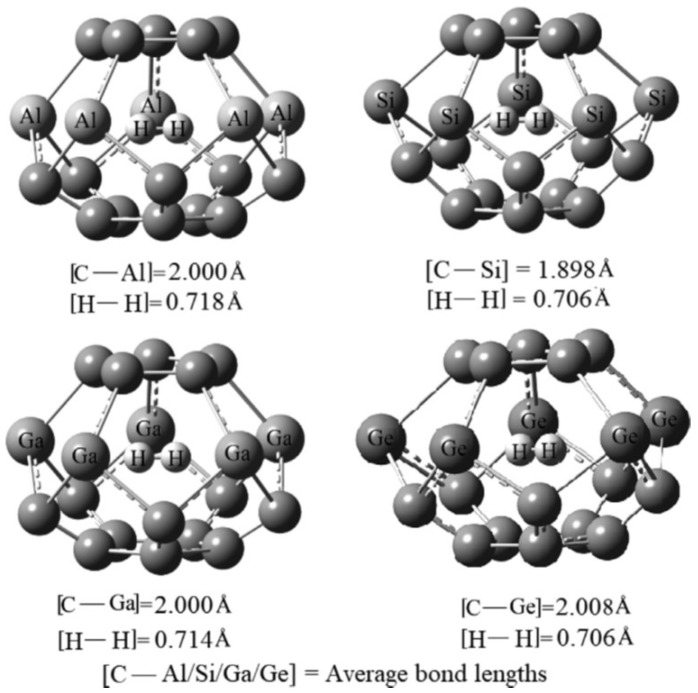
Optimized structures of H_2_ encapsulated doped clusters. Reprinted from [97], with permission from Elsevier.

To date, encapsulation inside a substitutionally Ge-doped fullerene has been examined in a single computational study: the investigation by Metin et al., who modeled H_2_ confinement within the C_15_Ge_5_ cage. Within the comparative series C_15_M_5_ (M = Al, Si, Ge, Ga), the Ge-containing cage displayed a favorable balance between structural integrity and dopant-induced distortion—more stable than Ga- and Al-doped analogs and only slightly less robust than Si (Figure 6). H_2_ encapsulation was thermodynamically favorable, with the fullerene maintaining its structural coherence and requiring only minimal local relaxation. The computed adsorption energy of 3.00 eV places C_15_Ge_5_ among the more effective hosts for small-molecule confinement [97].

Electronically, the Ge-doped cage responded strongly to the presence of H_2_. Encapsulation resulted in a measurable narrowing of the HOMO–LUMO gap, polarization centered on the dopant, and redistribution of charge density toward the cavity. This electronic sensitivity places Ge substitution in an intermediate regime -more responsive than Al or Ga doping yet less radical than Si—highlighting its ability to balance reactivity with mechanical resilience. The resulting interplay between cavity asymmetry, dopant effects, and internal polarization identifies C_15_Ge_5_ as a finely tuned system for endohedral modulation.

Beyond its intrinsic structural and electronic features, the behavior of C_15_Ge_5_ suggests several promising functional perspectives. The distinct changes in frontier orbitals and charge distribution induced by H_2_ encapsulation point to potential utility in molecular sensing, where guest uptake could be detected electronically or spectroscopically. Thermodynamically favorable confinement of small neutral molecules positions Ge-doped cages as candidates for storage or controlled release applications. Moreover, the asymmetric internal field generated by the germanium dopant underscores their relevance for molecular electronics and nanoscale functional devices, where internal charge redistribution can act as a tunable element.

### 4.4. Endohedral Complexes of Germanium Species Encapsulated in Pristine Carbon Fullerenes (Ge@C_n_)

Endohedral germanium–fullerene complexes (Ge@C_n_) form a structurally well-defined family in which one or more Ge atoms or small Ge clusters are confined inside an intact carbon cage. In contrast to substitutional heterofullerenes, the carbon framework remains chemically unmodified; germanium behaves strictly as an internal guest interacting with the delocalized π-electron cloud and curvature-induced σ-framework from within. This internal confinement perturbs symmetry, redistributes electron density, and modulates magnetic, mechanical, and spectroscopic properties, providing a controlled setting for probing how a metalloid center influences pristine fullerene cavities.

Computational investigations reported to date span a wide range of cage sizes and encapsulation patterns. Small strained cages such as C_20_ and C_28_ have been examined in detail, including Ge@C_28_ as a representative highly curved endohedral metallofullerene [98] and Ge@C_20_ within the group-14 E@C_20_ (E = Si, Ge, Sn, Pb) series [99]. For the archetypal C_60_ cage, diverse encapsulation scenarios have been explored: radiochemical formation of ^69^Ge@C_60_ and penetration pathways studied via ab initio MD [51], mechanical stabilization and compressive response analyzed through classical MD [100], and systematic DFT investigations of Ge_n_@C_60_ clusters containing up to nine Ge atoms [101,102,103]. Larger cages have also been addressed, including semiempirical analyses of Ge@C_80_ [104] and HF-level studies of Ge_n_@C_82_ with one to three internal Ge atoms [105].

The computational protocols across these studies cover essentially all major electronic-structure frameworks. Early work on C_28_ employed self-consistent Hartree–Fock with double-ζ basis sets and B-LYP DFT refinements implemented in TURBOMOLE [98]. Relativistic DFT using the PBE functional, ZORA Hamiltonian, STO-TZ2P Slater-type basis sets, and D3 dispersion in ADF was applied to the E@C_20_ series, with detailed energy-decomposition (EDA-NOCV), charge-flow, and TD-DFT spectral analyses [99]. For Ge_n_@C_60_ systems, most studies used GGA-PBE within SIESTA, Troullier–Martins pseudopotentials, and DZP basis sets to evaluate binding energies, HOMO–LUMO gaps, charge redistribution, magnetic moments, and reactivity descriptors [101,102,103]. Additional methodologies include classical MD with Tersoff-type bond-order potentials to probe mechanical resilience [100], ab initio LDA-based MD for implantation-driven encapsulation pathways [51], semiempirical AM1 modeling for Ge@C_80_ [104], and Hartree–Fock/STO-3G geometry analyses for Gen@C_82_ [105].

The scientific aims of these works converge on several key themes. A primary focus involves evaluating the thermodynamic feasibility and structural stability of Ge encapsulation across different cage sizes and encapsulation numbers [98,99,101,102,103,104,105]. A second major line of inquiry centers on the nature of bonding and electronic response—how internal Ge perturbs HOMO–LUMO gaps, local aromaticity, spin densities, charge distribution, and orbital symmetries relative to pristine fullerenes, frequently using methods such as EDA-NOCV, NIS, or Mulliken/spin-density mapping [98,99,101,102]. A third set of applications concerns functional behavior, including mechanical reinforcement under compression [100], magnetic tunability in Ge_n_@C_60_ clusters [101,102], and experimental feasibility of Ge@C_60_ formation through recoil implantation [51] or high-temperature arc-discharge methods [105]. Collectively, these studies delineate a size-dependent landscape in which encapsulation is least favorable in extremely strained small cages such as C_28_, becomes progressively more stabilized in C_20_ and C_60_, and leads to marked modulation of electronic, magnetic, and mechanical characteristics across the Ge@C_n_ family.

The most interesting applications of calculations in the study of these structures are presented below in more detail and Table 6. provides a summary of the computational protocols applied, specifying the semi-empirical methods, DFT functionals, and basis sets used in the respective studies on substitutional Ge-doped carbon fullerenes.

One of the earliest insights into this behavior came from Guo et al., who investigated a series of M@C_28_ complexes, including Ge@C_28_, as prototypes of highly strained endohedral metallofullerenes. Their analysis showed that inserting a germanium atom into the exceptionally curved C_28_ framework induces substantial geometric distortion, alters the topology of the frontier orbitals. These findings established C_28_ as a limiting case in which extreme curvature renders encapsulation energetically demanding and structurally disruptive, while simultaneously amplifying the sensitivity of the electronic structure to an internal guest. Comparable mechanistic insight into endohedral germanium was provided by Muñoz-Castro [99], who examined Ge@C_20_ within the group-14 E@C_20_ (E = Si, Ge, Sn, Pb) series. The study revealed a strong donor–acceptor interaction between Ge 4s/4p orbitals and the π-framework of the C_20_ cage, accompanied by distinct vibrational and optical signatures characteristic of metalloid–cage coupling under extreme curvature. The results demonstrated that Ge@C_20_ represents a characteristic case of curvature-driven orbital coupling, in which the geometric constraints of a highly strained cage enhance the electronic interactions between the encapsulated atom and the fullerene framework [98].

The archetypal fullerene C_60_ has received the most extensive attention. Ohtsuki and co-workers provided the first experimental confirmation of Ge@C_60_ formation by implanting radioactive ^69^Ge into fullerene films. Radiochemical detection, supported by ab initio molecular-dynamics simulations, showed that Ge atoms can penetrate the fullerene wall via ballistic recoil processes and become trapped inside the cage. This work demonstrated not only the feasibility of forming endohedral germanium complexes but also the mechanistic pathway by which encapsulation occurs under high-energy conditions. Further analysis of Ge@C_60_ properties was provided by Shen, who explored its mechanical behavior under compressive loading. Classical molecular-dynamics simulations revealed that an encapsulated Ge atom acts as an internal stabilizing element, enhancing the cage’s resistance to deformation and modifying its elastic response. This indicates that endohedral metalloid inclusion can reinforce the mechanical performance of carbon nanostructures, a feature relevant for designing fullerene-based materials with tunable mechanical resilience [51].

A series of detailed investigations by Umran and collaborators substantially expanded the understanding of size-dependent behavior in Ge_n_@C_60_ complexes. These studies, encompassing systems with up to nine encapsulated Ge atoms, established systematic trends in binding energies, electron affinities, charge redistribution, and magnetic moments. The results demonstrated that C_60_ can accommodate multiple Ge atoms while maintaining structural integrity, and that increasing guest multiplicity leads to progressive narrowing of the electronic gap and to the emergence of size-dependent magnetic characteristics. Complementary work using model potentials confirmed these trends and defined the geometric limits of fullerene encapsulation, suggesting that steric and electrostatic crowding inside C_60_ ultimately govern the maximum number of stable internal guests [101,102,103,104].

Larger cages have also been explored to assess how increased internal volume and altered surface topology affect germanium confinement. Türker’s study of Ge@C_80_ showed that encapsulated Ge can stabilize energetically less favorable C_80_ geometries and induce characteristic dipole-moment patterns across a series of group-IV endohedral analogs. Similarly, Roy and co-workers [105] examined Ge_n_@C_82_ (n = 1–3) and found that the elongated C_82_ cage enforces specific internal positions for Ge atoms, leading to characteristic axial distortions and monotonic reductions in the HOMO–LUMO gap as guest multiplicity increases. These results demonstrate that larger fullerenes provide a tunable environment in which anisotropy and internal free volume control the preferred binding sites and electronic responses of encapsulated germane species [104].

Together, these studies establish a coherent structure–property framework for endohedral Ge@C_n_ complexes. Encapsulation is least favorable in extremely strained small cages such as C_28_, becomes increasingly stabilized in C_20_ and C_60_, and acquires additional tunability in larger hosts such as C_80_ and C_82_. Across all cage sizes, germanium confinement induces characteristic reductions in band gaps, distinct charge-distribution patterns, and, in many cases, emergent magnetic behavior. These findings highlight the sensitivity of carbon nanocavities to metalloid encapsulation and underscore the potential of Ge-endohedral fullerenes as model systems for probing orbital confinement, and nanomechanical reinforcement in carbon-based materials.

In summary, the available studies establish a coherent structure–property relationship for endohedral Ge@C_n_ fullerenes, showing that curvature and internal free volume jointly govern the feasibility and consequences of germanium encapsulation. Highly strained cages such as C_28_ destabilize the confined atom, whereas C_20_ and C_60_ provide increasingly favorable environments in which electronic gaps narrow, charge redistribution intensifies, and magnetic responses emerge in a size-dependent manner. Larger hosts, including C_80_ and C_82_, introduce additional tunability by allowing anisotropic positioning and reduced steric crowding. Collectively, these findings highlight endohedral Ge complexes as versatile model systems for probing confinement-driven electronic modulation, nanoscale mechanical reinforcement, and metalloid-carbon interactions in pristine fullerene cavities, while revealing substantial opportunities for future exploration of their optical, magnetic, and device-relevant properties.

### 4.5. Exohedral Germanium Species Interacting with Pristine Carbon Fullerenes

Exohedral complexes formed between pristine carbon fullerenes and externally bound heteroatoms represent a distinct class of host-guest systems in which the fullerene cage remains chemically intact while an adsorbate interacts with its outer surface. In such architectures the curvature, π-electron distribution, and local electrostatic potential of the C_60_ cage determine the adsorption geometry and binding strength, while the adsorbate perturbs frontier orbital topology and induces localized charge redistribution. These systems are particularly suited for probing how main-group atoms engage in surface bonding to a conjugated carbon sphere, providing insight into reactivity gradients across the fullerene surface, preferential adsorption sites, and the balance between van der Waals and covalent contributions. Germanium, with its intermediate electronegativity and capacity for covalent bonding, serves as a valuable probe of exohedral reactivity in pristine fullerenes.

To date, only one quantum-chemical study has addressed exohedral germanium-C_60_ complexes at an atomistic and energetics-resolved level: the investigation by Nigam and Majumder [106]. Using density functional theory within the generalized-gradient approximation (PBE functional) and projector-augmented wave (PAW) pseudopotentials in a plane-wave basis, the authors performed full geometry optimizations and systematic energy-landscape scans for Ge approaching, adsorbing on, and attempting to penetrate the C_60_ cage. Their calculations established that Ge adsorption is strongly exohedral, with the ground-state configuration corresponding to Ge bonded covalently at a 6-6 ring bridge site, characterized by short Ge-C distances (~2.08 Å) and an interaction energy of −1.67 eV. Endohedral configurations were found to be energetically inaccessible, lying ~2 eV higher. Migration of Ge across the cage surface displayed a clear preference for low-coordination bridge sites, while radial insertion scans revealed a large penetration barrier (45–83 eV depending on trajectory), definitively excluding spontaneous encapsulation. The computational protocol encompassed geometry optimization (GO), binding-energy evaluation, and electronic-structure and property analysis (ESPA), including mapping of total-energy profiles along insertion pathways. Together, these results delineate germanium as a surface-bound, covalently interacting adsorbate whose preferred coordination reflects the local topology and π-electron density of C_60_ [106].

The electronic response of pristine C_60_ to Ge adsorption in this study highlights several functional implications. Bridge-site binding perturbs the local π-network and produces polarization localized around the adsorption site, affecting the HOMO–LUMO gap and suggesting that Ge adsorption could modulate fullerene-based electronic materials through controlled covalent surface functionalization. The substantial energy barriers for penetration imply that germanium cannot be introduced endohedrally under mild conditions, reinforcing the idea that Ge acts exclusively as a surface-binding species on pristine cages. The study therefore provides the first quantitative structure–energetics map for germanium adsorption on C_60_ and establishes a computational foundation for understanding exohedral main-group functionalization of fullerenes.

Two additional studies—Ohara et al. and Bertoni et al.—offer experimental context for Ge-C_60_ interactions, though they fall outside the computational scope of this section. Ohara’s work demonstrates that individual Ge atoms deposited on C_60_ tend to migrate and aggregate into small clusters (C_60_Ge_m_), whereas the study by Bertoni et al. investigates C_60_ adsorption on Ge(111) surfaces rather than germanium adsorption on fullerenes. Because neither study includes quantum-chemical calculations on Ge adsorbed to pristine C_60_, they are not incorporated into the analytical core of this computational chapter but serve as complementary evidence for exohedral (rather than endohedral) Ge-C_60_ behavior [53,107].

It should be emphasized that the results of density functional theory (DFT) calculations are sensitive to the choice of exchange–correlation (XC) functional. The widely used hybrid functional B3LYP was originally proposed by Becke and later combined with the Lee–Yang–Parr correlation term [108,109]. In contrast, PBE represents a generalized-gradient approximation functional formulated by Perdew, Burke and Ernzerhof [110,111], frequently applied in fullerene and heteroatom doping studies. For Ge-containing systems, hybrid functionals such as B3LYP, PBE0 and HSE06 are widely used due to their balanced description of electron correlation, structural parameters and HOMO–LUMO gaps. B3LYP typically provides reasonable geometries and energetics at moderate computational cost, while PBE0 and HSE06 offer improved band-gap prediction and often yield better performance for systems involving heavier main-group dopants. The choice of the optimal XC functional therefore depends on the targeted property—geometry optimization is commonly well reproduced by B3LYP/PBE0, whereas electronic excitations and accurate gap estimation may benefit from screened hybrids such as HSE06.

Relativistic contributions become increasingly important for Ge due to 4s–4p orbital contraction and spin–orbit interaction scaling approximately with Z^4^. For germanium-containing systems, relativistic effects may influence orbital energies, electron distributions and HOMO–LUMO gaps. In practical quantum-chemical simulations, these contributions can be incorporated at different levels of theory. The most common approach in DFT studies is the use of scalar-relativistic Hamiltonians such as the zeroth-order regular approximation (ZORA) or Douglas–Kroll–Hess (DKH), which account for mass-velocity and Darwin terms while retaining computational efficiency. Spin–orbit (SO) coupling, although typically small for structural optimization, can become relevant for excited-state and spectroscopic properties, and can be included via two-component relativistic DFT or SO-coupled pseudopotentials. In high-accuracy benchmark calculations, relativistic effective core potentials (RECP/ECP) or all-electron DKH basis sets are often applied to germanium as a heavier main-group element. These frameworks provide controllable inclusion of scalar and spin–orbit effects when needed.

## 5. Future Directions and Challenges

Although progress has been made in understanding germanium–fullerene chemistry, several key challenges remain unresolved. The successful isolation of substitutional heterofullerenes (C_59_Ge) continues to be a major synthetic barrier due to the high stability of pristine cages and the energetic cost of carbon replacement [39,70,79]. Future work may focus on template-assisted growth, high-pressure routes, or controlled ring-opening pathways to facilitate Ge insertion, building on strategies explored in related heterofullerene and metallofullerene synthesis [31,32,50,51,52]. Endohedral Ge@C_n_ structures, while spectroscopically suggested, still lack unambiguous crystallographic confirmation [31,32]. Advances in cryogenic ion-trapping, synchrotron-based diffraction, and soft-landing MS techniques could enable direct structural verification in the future.

Another promising direction is the exploration of mixed Si–Ge and multi-heteroatom systems, which may offer enhanced stability and tunability relative to mono-doped analogs [56,57,80,81]. From a computational perspective, machine-learning potentials, long-timescale molecular dynamics, and high-level correlated methods may refine energetic predictions and guide synthetic attempts. In addition to DFT, a variety of computational approaches have been employed to study germanium clusters. Early studies on small clusters (Ge_n_, n = 1–6) combined DFT geometry optimization with CCSD(T) single-point calculations to obtain reliable thermochemical and electron-affinity data [112]. For larger nanodots and cluster-derived materials, many-body techniques including GW and self-energy corrected methods have been applied to more accurately determine band gaps beyond standard DFT [113]. Alternative semi-empirical schemes such as transferable non-orthogonal tight-binding models have enabled efficient exploration of configurational spaces of medium-sized clusters [114].

We therefore note that while DFT remains the workhorse for modeling Ge–carbon and Ge–fullerene systems, benchmarking with higher-level methods or complementary approaches is advisable, especially where accurate binding energies or gap predictions are required [112,113,114,115]. Overall, progress will require the convergence of precise synthetic control, sensitive detection methods, and large-scale computational screening to guide feasible targets. A coordinated approach may ultimately enable the first robust experimental realization of substitutional and encapsulated Ge–fullerenes and unlock their potential in optoelectronics, catalysis, and molecular nanotechnology.

## 6. Conclusions

Germanium incorporation into carbon fullerenes—whether through substitution, exohedral complexation, or endohedral encapsulation—produces a consistent pattern of structural distortion, electronic polarization, and HOMO–LUMO gap modulation that distinguishes Ge-containing heterofullerenes from both pristine Cₙ cages and systems doped with lighter group-14 elements. Experimental studies have demonstrated that Ge can be introduced into fullerene matrices under high-energy recoil conditions and via arc-discharge synthesis, yielding mixtures that contain endohedral or defect-associated Ge species; however, the structural identity of substitutional Ge-fullerenes remains experimentally unresolved. In contrast, photochemical germylation reliably yields well-defined exohedral derivatives, clearly establishing germanium as a viable exohedral functionalization motif. Across all experimentally accessible classes, unambiguous structural authentication—particularly for substitutional Ge in C_60_—remains a key unmet challenge.

On the theoretical side, quantum-chemical methods have provided the most comprehensive insights into the feasibility, stability, and electronic consequences of germanium incorporation. DFT and ab initio studies agree that Ge substitution induces significant cage polarization, reduces symmetry, localizes frontier orbitals, and narrows band gaps, while maintaining the overall structural integrity of the fullerene framework even at elevated substitution levels. Computational work on exohedral and endohedral complexes shows that the presence of Ge creates preferential adsorption or coordination sites, enhancing guest binding energies and modifying optical and transport properties of the host cage. Altogether, the existing body of evidence positions germanium-doped fullerenes as electronically versatile nanostructures with promising functional characteristics, despite the limited experimental realization of structurally pure Ge-fullerene species.

## Figures and Tables

**Table 1:** Comparative summary of experimental investigations on germanium-fullerene systems.

Study	Synthesis Method	Analytical Techniques	Proposed Products/Findings	Limitations
Ohtsuki et al. [50]	Nuclear recoil implantation of Ge into C_60_/C_70_ mixed with GeO_2_ under deuteron irradiation	Gamma-ray spectroscopy; HPLC radiochemical separation, ab initio molecular dynamics simulations	Radiochemical evidence of Ge incorporation into fullerene—derived species; possible formation of Ge-containing heterofullerenes	Extremely low yield; no direct structural confirmation; identity of Ge location relies on simulations and radiochemical behavior
Ohtsuki & Ohno [51]	p- and d-induced recoil implantation on C_60_/GeO_2_ composites	Gamma-ray spectroscopy; radiochemical separation; classical molecular dynamics simulations	Incorporation of Ge into fullerene matrices; radiochemical signatures consistent with endohedral and/or defect-associated species	Very low product yield; unseparated mixture of species; lack of direct spectroscopic (NMR/MS/XRD) confirmation
Ohara et al. [53]	Dual-laser vaporization of Ge and C_60_	Photoelectron spectroscopy; mass spectrometry; DFT calculations	Formation of weakly adsorbed Ge_1-3_ clusters on C_60_ surface; no penetration of cage; exohedral interaction preferred	No endohedral or substitutional incorporation observed; gas-phase conditions; weak binding
Akasaka et al. [54]	Photochemical reaction of C_60_ with digermirane	UV-Vis; NMR; MS	First bis-germylation of C_60_; formation of 1,4-cycloadduct with two Ge-C bonds	Exohedral only; requires photolytic conditions; limited scalability
Kabe et al. [55]	Photochemical reaction of 1,2-digermacyclobutane with C_60_	UV–Vis; NMR; MS, PM3 semiempirical calculations	Formation of germylated derivative with 6,5- and 6,6-bridging units; closed-cage structure with Ge-C bridge	No crystallographic data; scope limited to specific cyclogermane precursors
Roy et al. [32]	Arc-discharge using composite GeO_2_ -graphite electrodes	UV–Vis; MALDI-TOF; FTIR; EDX; XPS	Formation of Ge@C_60_ and Ge@C_82_; spectroscopic confirmation of encapsulation (endohedral location).	Trace yields; unreacted GeO_2_ residues; difficulty in purification and quantification
Yılmaz & Uluğ (2005) [31]	Arc-discharge in He atmosphere using graphite tubes filled with elemental Ge	FTIR; FAB mass spectrometry; solvent extraction	Non-pristine fullerene-related species with mass patterns compatible with Ge-containing heterofullerenes	No structural assignment; mixed unidentified species; no elemental analysis; ambiguous Ge incorporation mode; FTIR dominated by C_60_/C_70_ bands
Saha et al. [52]	Heavy-ion recoil implantation into C_60_ thin films	Gamma-ray spectroscopy; radiochemical separation	High retention of radioactive ^69^Ge (~73%); evidence compatible with endohedral incorporation; quantitative uptake analysis	Formation yield extremely low (~10^−15^ g); co-existence of substitutional, endo- and exohedral species; no structural isolation
Kako et al. [56]	Photolytic bis-germylation of Lu_3_N@Ih-C_80_ with digermiranes	XRD; cyclic voltammetry; UV-Vis-NIR spectroscopy	Bis-germylated endohedral metallofullerene; altered redox potentials; shift in cage electron density	Synthetic yields moderate; limited to EMFs; requires controlled photolysis

NMR—nuclear magnetic resonance; TOF-MS—time-of-flight mass spectrometry; UV–Vis—ultraviolet–visible spectroscopy; FTIR—Fourier-transform infrared spectroscopy; Raman—Raman spectroscopy; XPS—X-ray photoelectron spectroscopy; ESR/EPR—electron spin resonance/electron paramagnetic resonance; MS—mass spectrometry.

**Table 2:** The point group (PG), sphericity parameter (SP), asphericity parameter (ASP), POAV, energy, energy with the zero-point correction (E-ZP), cohesive energy (E_coh_), and ΔE of C_20_ and the doped cages (SP in GHz-1, pi-orbital axis vector (POAV) in degree, energy and E-ZP in a.u., E_coh_ in eV/atom, and ΔE in eV).

Cage	PG	SP	ASP	POAV	Energy	E-ZP	E_coh_	ΔE
**C_19_B**	C_s_	0.026	0.005	22.81	−748.242	−748.132	6.134	0.280
**C_19_Al**	C_3v_	0.321	0.075	34.07	−965.803	−965.698	5.926	4.434
**C_19_Ga**	C_3v_	0.933	0.079	34.72	−2646.300	−2646.190	5.903	4.900
**C_20_**	C_2h_	0.122	0.015	22.90	−761.444	−761.331	6.148	0.000
**C_19_Si**	C_s_	0.380	0.079	33.80	−1012.830	−1012.730	5.960	3.755
**C_19_Ge**	C_s_	1.103	0.112	37.62	−2798.340	−2798.220	5.934	4.271
**C_19_N**	C_3v_	0.041	0.014	28.42	−778.090	−777.979	−0.352	−0.352
**C_19_P**	C_s_	0.540	0.118	38.61	−1064.760	−1064.650	6.102	0.913
**C_19_As**	C_3v_	1.207	0.129	41.19	−2957.170	−2957.060	6.080	1.352
**C_19_O**	C_s_	0.185	0.046	25.26	−798.484	−798.374	4.731	4.731
**C_19_S**	C_3v_	0.324	0.057	36.06	−1121.510	−1121.400	5.886	5.242
**C_19_Se**	C_3v_	1.054	0.085	38.17	−3122.720	−3122.610	5.894	5.070

Note; point group (PG), sphericity parameter (SP), asphericity parameter (ASP), pi-orbital axis xector (POAV), energy, energy with the zero-point correction (E-ZP), cohesive energy (E_coh_), and DE.

**Table 3:** Selected articles on application of theoretical calculations on substitutional Ge-doped carbon fullerenes.

Substitutional Ge-Doped C_60_ Fullerenes						
Ge-Doped Fullerene Derivative	Type of Calculation	Calculation Method		Computational Software	Aim of Study	First Author/Publication Year DOI (Ref)
		Semi Empirical	DFT (Functional & Basis Set)			
C_59_Ge	GO, ESPA, VFA, IR, NICS	-	B3LYP/6-31G(d), GIAO	Gaussian 09	Investigation of structural, electronic, vibrational, dielectric and aromatic properties changes in C_59_Ge relative to pristine C_60_ and C_60_ with other dopants (B, N, Al, As, P, Ga, Si)	Bai 2013 10.1155/2013/571709 [39]
C_59_Ge	GO, ESPA, IR	PM3	-	MOPAC 2002 via CAChe 1.33	Evaluation of structural, electronic and vibrational properties of Ge-doped C_60_ in comparison to pristine C_60_ C_59_Al and C_59_Si using semiempirical method	Ibrahim 2006 psroc.phys.ntu.edu.tw/cjp [69]
C_20-n_Ge_n_ (n = 1–4)	GO, ESPA	-	B3LYP/3-21G	Gaussian 09	Investigation of structural modifications, electronic properties, stability, and band gap evolution in Ge-doped C_20_ bowls with increasing dopant content for potential nanoelectronic and solar cell applications	Ajeel 2017 10.1016/j.cjph.2017.05.031 [75]
C_70-n_Ge_n_ (n = 1–3)	GO, ESPA, MD, Mdock	-	PBE-D	D-Mol^3^	Assessment of structural, electronic, dynamic, docking, and pharmacokinetic properties of Ge-doped C_70_ fullerenes in comparison with other semimetal- and nonmetal-doped systems for potential biomedical applications.	Alkhafaji 2024 10.1016/j.jorganchem.2024.123417 [77]
C_20-n_Ge_n_, (n = 1–5)	GO, VFA, ESPA	-	B3LYP, B3PW91, M06-2X, 6-311+G(d), 6-311++G(d,p), aug-cc-pVTZ	GAMESS	Investigation of structural, vibrational, electronic, and thermodynamic properties Ge-doped heterofullerenes to identify the most stable derivative and evaluate its potential for hydrogen storage and electronic applications	Baei 2018 10.1002/hc.21410 [60]
C_19_Ge	GO, VFA, ESPA	-	B3LYP, 6-31G(d)	Gaussian 09	Investigation of structural distortions, stability, electronic properties, and aromaticity of C_19_Ge in relation to pristine C_20_ and C_20_ with other dopants (B, N, O, Al, S, P, Ga, Si, As, Se)	Bai 2014 10.1080/1536383X.2013.863762 [72]
C_20_-C_16_Ge_4_	GO, ESPA, IV	EHT	-	Atomistix ToolKit /Virtual NanoLab	Investigation of electronic transport, transmission spectra, and conduction properties in Ge-doped double C_20_-cage molecular junction compared to other substitutional dopants (Si, Sn, Pb)	Kaur 2017 10.1007/s00894-017-3430-9 [82]
C_59_Ge, C_58_Ge_2_	GO, ESPA	AM1	-	HyperChem 7.0.	Investigation of structural, electronic, and nonlinear optical properties of Ge-doped fullerenes for photonic and optoelectronic applications	Kityk 2004 10.1016/j.matlet.2003.10.039 [78]
Si_4_Ge_4_C_12_	GO, ESPA, NICS	-	B3LYP/6-311+G(d), B3LYP/aug-cc-pVTZ, B3LYP/6-311++G(d,p)	GAMESS	Investigation of structural, electronic, vibrational, and stability properties of heterofullerenes (including Ge substitution) to evaluate their potential for hydrogen storage and optoelectronic applications	Koohi 2018 10.1007/s11224-017-1071-3 [80]
Si_n_Ge_n_C_20-2n_ (n = 1–10)	GO, VFA, ESPA	-	B3LYP/6-311+G(d), B3LYP/6-311++G(d,p), M06-2X/6-311++G(d,p), B3PW91/6-311+G(d), B3LYP/aug-cc-pVTZ	Gaussian 98	Investigation of stability, aromaticity, and electronic properties of Si-Ge substituted C_20_ heterofullerenes as candidates for hydrogen storage and optoelectronic applications	Koohi 2020 10.1007/s00706-020-02596-4 [81]
C_20-n_Ge_n_ (n = 5–10)	GO, VFA, ESPA	-	B3LYP/6-31+G(d), B3LYP/6-311++G(d,p), B3LYP/aug-cc-pVTZ	Gaussian 98	Investigation of structural, electronic, and stability properties of Ge-doped C_20_ analogs to evaluate their aromaticity and potential for hydrogen storage applications	Koohi 2015 10.1007/s00706-014-1388-1 [74]
C_14_Ge_6_	GO, VFA, ESPA, NICS	-	B3LYP/6-311+G(d), B3LYP/6-311++G(d,p), B3LYP/aug-cc-pVTZ	GAMESS	Investigation of structural stability, bonding patterns, and electronic properties of C_14_Ge_6_ heterofullerenes to identify stable isomers with potential for hydrogen storage applications	Koohi 2017 10.1002/poc.3678 [76]
C_58_Ge	GO, VFA, ESPA	-	B3LYP/6-31G, B3LYP/6-31G(d)	Gaussian 98	Investigation of structural stability, electronic properties, and charge distribution of the novel [5,6]-heterofullerene-like C_58_Ge as a stable chiral derivative with distinct features from pristine C_60_	Liu 2007 10.1039/b704510g [79]
C_12_Ge_8_	GO, ESPA, VFA	-	B3LYP/6-31+G(d), MP2/6-311+G(d)	Gaussian 98	Assessment of stability and electronic properties of C_12_Ge_8_ compared with B-, Al-, Si-, Ga-, As-, N- and P-doped analogs for hydrogen storage applications	Naderi 2012 10.1007/s11224-012-9958-5 [73]
C_59_Ge, C_69_Ge	MD, ESPA	-	Ab initio LDA	In house code	Assessment of synthesis and stability of Ge-doped C_60_ using radiochemical methods and ab initio MD simulations	Ohtsuki 1999 10.1103/PhysRevB.60.1531 [50]
C_69_Ge	GO, ESPA, SRCM	-	B3LYP/6-31G(d)	Gaussian, CHIMEA	Investigation of the stability, chirality, and spectroscopic features of Ge-substituted C_69_ heterofullerenes compared with B, Si, N, P, and As analogs	Ostrowski 2013 10.1016/j.tetasy.2013.07.022 [71]
C_59_Ge	GO, ESPA	-	B3LYP/SDDALL	Gaussian 98/03	Investigation of structural and electronic properties of C_59_Ge and related substitutional heterofullerenes (C_59_Si, C_59_Sn, C_59_B, C_59_N, C_59_P) to evaluate the effect of group-IV substitution on cage distortion, charge transfer, dipole moment, and HOMO–LUMO gap.	Simeon 2005 10.1002/qua.20718 [70]

GO—geometry optimization, VFA—vibrational frequency analysis, ESPA—electronic & structure properties, IV—Current–Voltage characteristics, SRCM—structure related chirality measurements.

**Table 4:** Selected articles on application of theoretical calculations on exohedral complexes of substitutional Ge-doped carbon fullerenes.

Exohedral Complexes of Substitutional Ge-Doped Carbon Fullerenes							
Ge-Doped Fullerene Derivative (Host)	Adsorbed Guest	Type of Calculation	Calculation Method		Computational Software	Aim of Study	First Author/Publication Year DOI (Ref)
			Semi Empirical	DFT (Functional & Basis Set)			
C_59_Ge	amphetamine	GO, ESPA, E_ads_ (with BSSE), NICS	-	B3LYP-D, 3-21G(d), 6-31G, 6-311++G(d,p), GIAO	GAMESS	Investigation of adsorption process and aromaticity response of Ge-doped C_60_ as a molecular sensor for amphetamine (in comparison to C_59_B, C_59_Ga, C_59_, C_59_Al, and pristine C_60_)	Bashiri 2017 10.1016/j.vacuum.2016.12.003 [85]
C_59_Ge	amantadine	GO, ESPA, E_ads_ (with BSSE), IR, NMR, PCM	-	B3LYP/6-31G(d), B3LYP/cc-pVDZ	Gaussian 09	Investigation of noncovalent interactions between Ge-doped C_60_ and amantadine in gas and aqueous phases (in comparison to C_59_B, C_59_Ga, C_59_Si, C_59_Al, and pristine C_60_)	Parlak 2017 prim 10.1016/j.cplett.2017.04.025 [83]
C_59_Ge	amantadine	GO, ESPA, E_ads_	-	HSEH1PBE/6-311G(d), B3LYP-D3/6-311G(d), wB97XD/6-311G(d), M062X/6-311G(d)	Gaussian 16	Assessment of adsorption strength and electronic reactivity descriptors for amantadine on Ge-doped C_60_ (in comparison to C_59_B, C_59_Ga, C_59_Si, C_59_Al, C_59_P, C_59_As, C_59_N and pristine C_60_)	Mohammadi 2021 10.1142/S2737416521500022 [84]
C_59_Ge	carbamazepine	GO, ESPA, E_ads_ (with BSSE), PCM	-	ωB97XD/6-31G(d), M06L/6-31G(d)	Gaussian 09	Investigation of interaction energy, solvent effects, and electronic properties of carbamazepine adsorbed on Ge-doped C_60_ (in comparison to C_59_B, C_59_Ga, C_59_Si, C_59_Al, C_59_P, C_59_N and pristine C_60_)	Silva 2022 10.1038/s41598-022-19258-6 [90]
C_59_Ge	methadone	GO, ESPA, E_ads_ (with BSSE), TDDFT	-	M062X/6-31G(d), B3LYP/6-31G(d)	GAMESS	Investigation of structural, spectroscopic and electronic response of Si-doped C_60_ to methadone adsorption using TDDFT and quantum reactivity descriptors (in comparison to C_59_B, C_59_Si and pristine C_60_)	Hoseininezhad-Namin 2023 10.1007/s00894-023-05470-2 [94]
C_59_Ge	epigallocatechin-3-gallate (EGCG)	GO, ESPA, E_ads_ (with BSSE)		B3LYP-D3/6-31G(d)	Gaussian 16	Analysis of non-covalent interactions and electronic reactivity in EGCG adsorption on Ge-substituted C_60_ (in comparison to C_59_B, C_59_Ga, C_59_Si, C_59_Al, C_59_P and pristine C_60_)	Singh 2025 10.1016/j.comptc.2024.115001 [92]
C_59_Ge	serine	GO, ESPA, E_ads_ (with BSSE)	PM6	PBE0/6-311G(d), B3LYP-D3/6-311G(d), ωB97XD/6-311G(d), M06-2X/6-311G(d)	Gaussian 16	Investigation of structural, electronic, and interaction properties of serine adsorption on Ge-doped C_60_ compared to Si-doped and pristine analogs for potential nanocarrier and nanosensor applications	Mohammadi 2021 prim 10.1007/s12633-021-01408-6 [86]
C_59_Ge	cysteine	GO, ESPA, E_ads_ (with BSSE)	PM6	PBE0/6-311G(d), B3LYP-D3/6-311G(d), ωB97XD/6-311G(d), M06-2X/6-311G(d)	Gaussian 16	Investigation of adsorption properties and interaction mechanisms of cysteine on Ge-doped C_60_ compared to Si-doped and pristine analogs	Mohammadi 2021 bis 10.1007/s00894-021-04960-5 [87]
C_59_Ge	acrolein	GO, ESPA, E_ads_ (with BSSE)	PM6	PBE0/6-311G(d), B3LYP-D3/6-311G(d), ωB97XD/6-311G(d), M06-2X/6-311G(d)	Gaussian 16	Investigation of intermolecular interactions, structural, electronic, and adsorption properties of Ge-doped C_60_ with acrolein (compared with C_59_Si and pristine C_60_)	Mohammadi 2022 10.1007/s11224-021-01847-2 [89]
C_59_Ge	hexachlorobenzene (HCB)	GO, ESPA, E_ads_ (with BSSE)	PM6	PBE0/6-311G(d), B3LYP-D3/6-311G(d), ωB97XD/6-311G(d), M06-2X/6-311G(d)	Gaussian 16	Investigation of adsorption behavior, structural and electronic modifications, and interaction mechanisms of HCB on Ge-doped C_60_ compared to pristine and Si-doped analogs for potential pollutant removal applications	Mohammadi 2023 10.1016/j.chphi.2023.100234 [88]
C_59_Ge	letrozol	GO, ESPA, E_ads_ (with BSSE), PCM	-	B3PW91, 6-311G(d,p)	Gaussian 03	Investigation of adsorption behavior, electronic properties, solvation effects, and drug delivery potential of letrozole on Ge-doped C_60_ (compared with pristine C_60_ and C_59_Si)	Behmanesh 2020 10.1007/s00706-019-02524-1 [91]
C_55_Ge_5_	Li	GO, ESPA	MNDO	-	GAMESS	Investigation of stability, electronic structure, spin density localization, Li-fullerene coordination of C_55_Ge_5_ (compared with C_55_Si_5_, C_55_Sn_5_, C_55_B_5_, C_55_Al_5_, C_55_N_5_, C_55_P_5_).	Chistyakov 1998 10.1023/A:1016016329822 [95]
Si_11_GeC_12_	caffeine	GO, ESPA, E_ads_ (with BSSE), TDDFT PCM	-	B3LYP/6-311G(d,p)	Gaussian 09	Investigation of caffeine adsorption on Ge-doped heterofullerene Si_11_GeC_12_, with comparative analysis of Si_12_C_12_ and Si_11_BC_12_ analogs for sensing applications	Mahdi 2024 10.1016/j.molliq.2024.124467 [96]
C_59_Ge	ferulic acid	GO, ESPA, E_ads_ (with BSSE)	-	B3LYP-D3/6-31G(d)	Gaussian 16	Investigation of adsorption behavior, charge transfer, and electronic properties of Ge-doped C_60_ interacting with ferulic acid (a pharmacologically active compound) to assess its potential as a reusable biosensor.	Singh 2025 10.1016/j.comptc.2024.114998 [93]

Abbreviations used in Table 4: GO—geometry optimization; ESPA—electronic structure and property analysis; Eads—adsorption energy; BSSE—basis set superposition error correction; IR—infrared spectroscopy simulation; NMR—nuclear magnetic resonance shielding calculations; NICS—nucleus-independent chemical shift (aromaticity index); PCM—polarizable continuum solvation model; TD-DFT—time-dependent density functional theory (optical properties); Semi-empirical methods: PM6—Parametric Method 6; MNDO—Modified Neglect of Diatomic Overlap. DFT functionals and basis sets used: B3LYP, B3LYP-D, B3LYP-D3—Becke three-parameter hybrid functional with (D/D3) dispersion correction; HSEH1PBE—screened hybrid Heyd–Scuseria–Ernzerhof functional; ωB97XD—long-range corrected hybrid functional with empirical dispersion; M06-2X / M06L—Minnesota functionals for main-group chemistry (double-hybrid / local); PBE0—Perdew–Burke–Ernzerhof hybrid functional; B3PW91—Becke three-parameter functional with Perdew–Wang correlation; Basis sets: 3-21G(d), 6-31G, 6-31G(d), 6-311G(d), 6-311++G(d,p), cc-pVDZ—standard split-valence basis sets used for organic systems; GIAO—Gauge-Independent Atomic Orbital scheme for magnetic shielding calculations.

**Table 5:** Computed parameters for heterofullerenes [94].

Name	E_ad_	E_BSSE_	D	Q_NBO_	E_HOMO_	E_LUMO_	E_g_	%Δσ	DM	A	I
**Methadone**					−5.68	−0.65	5.03		2.66	5.68	0.65
**C_60_**					−5.99	−3.22	2.76		0	5.99	3.22
**A**	−0.22	0.17	3.08	0.01	−5.73	−3.07	2.66	−3.91	4.22	5.73	3.07
**B**	−0.25	0.17	2.83	0.007	−5.66	−3.25	2.41	−12.7	2.21	5.66	3.25
**GeC_59_**					−5.89	−3.77	2.12		0.33	5.89	3.77
**C**	−1.21	0.36	1.94	0.206	−4.62	−2.28	1.77	−16.58	16.32	4.62	2.28
**D**	−2.08	0.33	2.11	0.286	−4.64	−2.58	2.06	−2.73	12.66	4.64	2.58
**SiC_59_**					−5.82	−3.17	2.65		0.82	5.82	3.17
**E**	−1.23	0.2	1.81	0.231	−4.61	−2.86	1.75	−19.32	17.11	4.61	2.86
**F**	−1.26	0.21	1.99	0.265	−4.67	−2.58	2.09	−3.83	12.6	4.67	2.88
**BC_59_**					−5.66	−3.22	2.44		0.37	5.66	3.22
**G**	−0.54	0.2	1.61	0.299	−4.77	−2.67	2.1	−13.7	12.01	4.77	2.67
**H**	−0.71	0.28	1.7	0.377	−4.83	−2.75	2.08	−14.66	8.43	4.83	2.75

Adsorption energy (E_ads_/eV), basis set superposition error energy (E_BSSE_/eV), bond distance between methadone and fullerenes (D/Å), NBO charge on the methadone in systems (Q_NBO_/e), HOMO energy (E_HOMO_/eV), LUMO energy (EL_UMO_/eV), energy gap (E_g_/eV), %Δσ change in electrical conductivity after the methadone adsorption, dipole moment in gas phase (DM/debye), electron affinity (A), and ionization potential (I).

**Table 6:** Selected articles on application of theoretical calculations on endohedral complexes of pristine carbon fullerenes with encapsulated Ge atoms or clusters.

Endohedral Complexes of Carbon Fullerenes with Ge Atoms or Clusters							
Fullerene Type (host)	Guest	Type of Calculation	Calculation Method		Computational Software	Aim of Study	First Author/Publication Year DOI (Ref)
			Semi Empirical	DFT (Functional & Basis Set) or HF			
C_20_	Ge	GO, EDA-NOCV ESPA, TDDFT	-	PBE-D3/STO-TZ2P (ZORA)	ADF	Investigation of the structural stability, electronic properties, and bonding mechanism of Ge@C_20_ in comparison with other group-14 endohedral analogs (Si@C_20_, Sn@C_20_, Pb@C_20_)	Muñoz-Castro, 2017 10.1002/jcc.24809 [99]
C_60_	Ge_n_ (n = 1–4)	GO, ESPA, E_ads_	-	PBE/DZP	SIESTA	Investigation of structural stability, binding energy, electron affinity, and magnetic behavior of endohedral Ge_n_@C_60_ complexes to determine limits of encapsulation and magnetic moment trends with increasing Ge content	Umran 2013 10.1063/1.4791012 [101]
C_60_	Ge_n_ (n = 1–9)	GO, ESPA, E_ads_	-	PBE/DZP	SIESTA	Investigation of the structural stability, binding energy, electronic and magnetic properties of endohedral Ge_n_@C_60_ complexes, including limits of encapsulation and comparison with Si_n_@C_60_ analogs	Umran 2014 10.1016/j.physb.2013.12.039 [102]
C_60_	Ge_n_ (n = 1–4)	GO, ESPA, E_ads_	Model potentials (Brenner, Lennard–Jones, Gupta) *	-	Custom code	Investigation of structural stability, binding energy, and geometric limits of Ge_n_@C_60_ endohedral complexes using empirical model potentials, with comparison to Si-, Au-, and Tl-doped analogs	Umran 2015 10.1088/2053-1591/2/5/055603 [103]
C_82_	Ge_n_ (n = 1–3)	GO, ESPA	-	RHF/UHF, STO-3G	GAMESS	Investigation of structural and electronic properties of Ge@C_82_ endohedral fullerenes with varying Ge content to assess stability and band gap trends	Roy 2006 10.1080/15533170500471532 [105]
C_80_	Ge	GO, ESPA	AM1	-	HyperChem, ChemPlus	Investigation of the structural and electronic properties of Ge@C_80_ and related M@C_80_ (M = Be, C, Si) endohedral fullerenes to evaluate stability, dipole moment trends, and HOMO–LUMO characteristics across group-IV dopants	Türker 2003 10.1016/S0166-1280(03)00084-8 [104]
C_28_	Ge	GO, ESPA	-	HF/DZ, B-LYP/DZ	TURBOMOLE	Early prediction of structure, stability, HOMO–LUMO gap and energetics of Ge@C_28_ among M@C_28_ metallofullerenes	Guo 1993 10.1063/1.465758 [98]
C_60_	^69^Ge	GO, ESPA, AIMD	-	LDA (mixed-basis) AIMD	Custom code	Experimental formation of Ge@C_60_ via recoil implantation and AIMD modeling of penetration and trapping energies	Ohtsuki 2004 10.1103/PhysRevLett.93.112501 [51]
C_60_	Ge	MD, ESPA	-	Tersoff-type bond-order potentials	Custom MD code	Evaluation of mechanical response, structural deformation, and reinforcement effects in Ge@C_60_ under hydrostatic and uniaxial compression compared with pristine C_60_	Shen 2006 10.1016/j.chemphys.2006.04.004 [100]

* empirical calculations EDA-NOCV—Energy Decomposition Analysis with Natural Orbitals for Chemical Valence, ADF—Amsterdam Density Functional, AIMD—ab initio molecular dynamics.

## Data Availability

No new data were created or analyzed in this study. Data sharing is not applicable to this article.
